# Upcoming multi-visceral robotic surgery systems: a SAGES review

**DOI:** 10.1007/s00464-024-11384-8

**Published:** 2024-11-14

**Authors:** Ankit Sarin, Sarah Samreen, Jennifer M. Moffett, Edmundo Inga-Zapata, Francesco Bianco, Nawar A. Alkhamesi, Jacob D. Owen, Niti Shahi, Jonathan C. DeLong, Dimitrios Stefanidis, Christopher M. Schlachta, Patricia Sylla, Dan E. Azagury, for The SAGES Robotic Platforms Working Group

**Affiliations:** 1 University of CA – Davis Health, 6th Floor, 2335 Stockton Blvd., Sacramento, CA 95817 USA; 2 University of Texas Medical Branch, Galveston, TX USA; 3 Surgical Research Lab, Larkin Health System, Miami, FL USA; 4 Facultad de Medicina Humana, Universidad de San Martín de Porres, Lima, Peru; 5 University of Illinois at Chicago, Chicago, IL USA; 6 Western University and London Health Sciences Centre, London, ON Canada; 7 ECU Health Medical Center, Greenville, NC USA; 8 University of Colorado Anschutz Medical Campus, Aurora, CO USA; 9 University of Tennessee Medical Center, Knoxville, TN USA; 10 Indiana University School of Medicine, Indianapolis, IN USA; 11 Schulich School of Medicine and Dentistry, Western University, London, ON Canada; 12 Mount Sinai Health System, New York, NY USA; 13 Stanford University School of Medicine, Stanford, CA USA

**Keywords:** Robotic surgery, Innovation, Robotic platforms, Surgical architecture, Telemedicine, Imaging

## Abstract

**Background:**

Robotic surgical procedures continue to increase both in the United States (US) and worldwide. Several novel robotic surgical platforms are under development or undergoing regulatory approval. This review explores robotic platforms that are expected to reach US consumers within the next 2–3 years.

**Methods:**

The SAGES Robotic Platforms Working Group identified robotic surgery platforms in various stages of development and selected multi-visceral systems nearing or completing the US Food and Drug Administration (FDA) approval process. We outline key system components including architecture, unique features, development status, regulatory approval, and expected markets.

**Results:**

We identified twenty robotic platforms that met our selection criteria. Ten companies were based in North America, and ten were based in Europe or Asia. Each system is described in detail and key features are summarized in table form for easy comparison.

**Conclusion:**

The emergence of novel robotic surgical platforms represents an important evolution in the growth of minimally invasive surgery. Increased competition has the potential to bring value to surgical patients by stimulating innovation and driving down cost. The impact of these platforms remains to be determined, but the continued growth of robotic surgery seems to be all but assured.

Surgical robots have come a long way since the first reported human use in 1985 [1] Traditionally, robotic-assisted surgery was thought of as a tool for minimally invasive surgery, but as the technology developed with more sophisticated instruments and workflows, so too did the complexity of the procedures that could be offered. In fact, a terminology change to minimal access surgery has been proposed to account for the complexity of the robotic procedures that are now performed. Increasingly, through integration of smart technology, new generation robotic surgery platforms are seen to have the potential to disrupt and transform workflows in broad areas including clinical practice, surgical education, and data capture. Intuitive’s da Vinci robot has dominated the market since its first FDA approval for abdominal surgeries in 2000 [2]. However, in the past decade, the number of robotic surgery platforms under development has rapidly increased [3]. The scale of these projects has ranged from small start-up companies to large scale MedTech giants. This increasing competition is expected to lower the cost of technology, hasten engineering developments, and lead to rapid market penetration.

It is important to note that the term “*robot*” is a misnomer when applied to surgical robots since currently there is not any commercially available *fully* autonomous surgical multi-visceral robot. Currently available surgical robots insert a computer interface between the surgeon and the patient, assuming some degree of control that was previously reserved for the surgeon [4], allowing for enhancements that include advanced visualization, improved dexterity, different ergonomics, and integration of artificial intelligence.

For this review, we explore emerging surgical robotic platforms designed for multi-visceral surgery that seek to enter the US market. To frame this paper, we start with some specifications. These include platform autonomy, which refers to the degree to which the robotic system can perform tasks independently or assist the surgeon with decision-making and movements. Platform and arm architecture pertains to the structural design of the robotic system, including the arrangement and functionality of its arms. Instrument design covers the creation of specialized tools that the robotic arms use, designed for specific surgical tasks to enhance dexterity and precision. The optic system includes an overview of the cameras and imaging technology used by these systems for accurate navigation. The console is the interface where the surgeon controls the robotic system. Surgical sustainability refers to the ability to lower the environmental impact of surgical operations, including waste reduction, emissions minimization, energy conservation, and the promotion of sustainable procurement and disposal methods. Telesurgery is surgical technique that allows a surgeon to perform surgery on a patient remotely using robotic systems and wireless networking. The regulatory approval process is the series of evaluations overseen by the FDA before the platform reaches consumers and market strategy is an overview of how the robotic company will articulate its value proposition to its customers. We then describe the design, features, and potential future implications of individual systems.

## Specifications

### Platform autonomy

Within the framework of autonomy there are three main types of robotic systems: active systems which work autonomously to complete pre‑programmed tasks, semi‑active systems which allow for a surgeon‑driven element to complement the pre‑programmed element, and finally systems which are entirely dependent on surgeon activity. Most abdominal robotic systems currently approved by the FDA are essentially robotically assisted telemetry manipulation devices [5]. Their degree of automation is minimal. Even within this paradigm, however, surgeon control over the system is variable with several robotic platforms using finger actuators and pedals to control the movement of the robot while some, such as Moon Surgical, require direct manual control by the surgeon [6].

### Platform architecture

Platform architecture refers to the overall arrangement of the robotic system as it interacts with patients, surgeons, and support staff. Generally, the robotic platforms have three components; a set of arms that interact with the patient (patient cart), a component that accepts input from the surgeon (surgeon cart), and a processing unit that manages the computing resources (the tower). It is possible to consolidate two of these three components into just one, so they only have two components, potentially reducing the space required in the operating room or in transportation. The Moon surgical system combines surgeon input with the arms through direct manual control. Similarly, the computing resources are housed in the surgeon console in the Senhance system. Another unique approach is to combine one of these units with existing OR resources such as for the Ottava system where the robotic arms are being incorporated into the operative bed.

### Arm and instrument architecture

The robotic arms used for surgical handling of tissue vary across systems. Systems can be classified into a) Single arm systems that are designed to work either transabdominally (Virtual Incision—MIRA or Vicarious Beta) or trans luminally (Flex); or b) Multi-arm systems which are for more general purpose. The multi-arm systems tend to be modular with 3 to 5 arms which are either mounted on individual carts (Asensus, CMR Versius, SS Innovation, Hugo) or boom-mounted (DaVinci, Avetera, Hintori) with the exception of Ottava, which is being designed to be bed-mounted. At the level of the wrists, most arms have at least 7 degrees of freedom with some such as CMR Versius having the capability for 360° rotation. Most platforms have proprietary instruments with either single use (Avetera) or multi use (SS Innovation). 8 mm instruments are the standard with some exceptions such as Asensus offering 5 mm instruments and 3 mm instruments for pediatric patients. Most offer monopolar and bipolar energy options although stapling technology and advanced energy are less ubiquitous across platforms.

### Optic systems

Three‑dimensional visualization in surgery has demonstrated potential benefits to operative planning, procedure performance, surgical skill acquisition, and patient outcomes. Comparison of 2D–3D vision during performance of Fundamentals of Laparoscopic Surgery tasks demonstrates reduced time to task completion and improved ease and efficiency of task performance [7]. Most robotic surgical platforms offer 3D visualization, which allows for improved proficiency with greater speed to task completion and decreased errors [7].

Many robotic surgical platforms also incorporate near‑infrared‑emitting light sources in conjunction with near‐infrared cameras as part of their visualization portfolio. This allows the use of fluorescence to identify anatomic structures or evaluate tissue perfusion during surgery, using fluorophores. Some studies have proposed improved clinical outcomes with the use of ICG including improved operative time. Rates of conversion, length of stay and even mortality [8, 9]. ICG has also been used to visualize and quantify bowel perfusion of colorectal anastomoses, with some studies demonstrating decreased anastomotic leak rates using this technique [10]. For example a randomized, open-label, phase 3, trial in Japan (EssentiAL trial) showed statistically significant reduction in anastomotic leak and reoperation rates after low rectal cancer resections with the use of ICG [11].

### Surgeon console

Robotic surgery platforms have either closed or open consoles. In a closed console system, the surgeon fixes their head position for viewing, leading to a standard field of view. It also allows for an immersive experience. In an open console system, traditional screens assisted with 3-d glasses are used. The surgeon can move their head freely during the operation, and although head motion may be associated with decreased efficiency, open console systems can allow for improved surgeon communication with team members.

### Surgical sustainability

There has been call for environmental sustainability in operating rooms by multiple organizations including American College of Surgery [12] as well as The Society of American Gastrointestinal and Endoscopic Surgeons (SAGES) and the European Association for Endoscopic Surgery (EAES) [13]. For Robotic Systems use of multiuse rather than single use instruments and ability to work across specialties and operative environment results in higher sustainability. Given its importance we have rated robotic systems as high or low in sustainability based on reusability of system components.

### Telesurgery

The ability to perform surgery on a patient remotely using robotic systems and wireless networking. Since this first telerobotic surgery in 2001, the development of remote surgery has been difficult because of significant limitations of the network system. While robotic platforms routinely use communication through cables between the master controllers on the console and the robotic arms longer distance communication has been a challenge. Time-lag refers to the delay in transferring sensory and motor modalities between two far-reaching locations. Increased latency can increase the chances of inaccuracies, along with a lengthy operation.

### Regulatory approval process

To date, some robotic platforms have obtained FDA clearance through the 510 k pathway, which grants approval to market the platform for a certain indication. It is common for companies to go through this process multiple times over, either to obtain additional indications for use, or clearance related to a modified version of the platform. When available, we provide approval status, indication for use, if approved, or estimated timeframe of FDA clearance. If the platform has already obtained approval for use in Europe, we provide CE mark status.

### Market strategy

Market strategy is a challenging topic to report. This often involves highly confidential strategies that companies are not willing to disclose publicly, especially prior to their market availability. Financial information is also not comparable between systems since cost and pricing information, even when available, is confounded by differences in monthly operating costs, support fees, instrument costs as well as other payment models such as leasing.

## IDEAL framework

Given the rapid development of robotic platforms worldwide, it is important ensure that surgical robots are developed and evaluated with the highest standards of safety, effectiveness, and ethical considerations. The IDEAL framework [14] offers a structured pathway for the evaluation of surgical robotics, balancing innovation with rigorous assessment to maximize patient and societal benefits. It underscores the importance of transparency, collaboration, and the need to address global health inequities and sustainability in surgical robotics. Under this framework surgical robotics are assigned the following stages:

Preclinical and Early Clinical Stages (IDEAL Stages 0, 1, 2a):

- Focuses on ensuring the safety, feasibility, and acceptability of surgical robots through rigorous testing.
- Recommends standardizing publications of technical and clinical data, ensuring transparent documentation of device changes, and systematically evaluating AI-integrated systems.
- Highlights the importance of clinician training, patient-centered consent processes, and early economic modeling.

Comparative Evaluation (IDEAL Stages 2b and 3):

- Emphasizes the need for robust comparative studies to evaluate the effectiveness of surgical robots against current best practices.
- Recommends using validated clinical and technical outcomes, considering learning curves, and ensuring transparent and independent data collection and analysis.
- Encourages the exploration of patient acceptability and long-term economic impact, particularly in low-resource settings.

Long-Term Monitoring (IDEAL Stage 4):

- Focuses on continuous performance monitoring in real-world settings to ensure ongoing safety and effectiveness.
- Recommends the use of real-world data (RWD) for high-quality, transparent evaluations and emphasizes the need for international collaboration to create comprehensive datasets.
- Advocates for the inclusion of environmental impact assessments and sustainability considerations in long-term evaluations.

## Upcoming robotic platforms

We include systems with origins in North America (Table 1) Europe (Table 2) and Asia (Table 3).

**Table 1:** Upcoming North American abdominal surgical robotic systems

	Senhance	Endoluminal Surgical ELS	Ottava	MARS	Flex Robotic system	Hugo	Maestro	Enos	Vicarious	MIRA
Manufacturer	Asensus Surgical	Endoquest Robotics	Johnson & Johnson	Levita Magnetics	Medrobotics Corp	Medtronic	Moon Surgical	Titan Medical	Vicarious	Virtual Incision
Platform	Senhance	Endoluminal Surgical (ELS)	Ottava	MARS	Flex Robotic System	Hugo	Maestro	Enos	Beta 2	MIRA
Country of origin	USA— Durham, NC	USA—Houston, TX,	USA	USA	USA	USA	USA	Canada—Toronto	USA—Waltham, MA	USA—Nebraska
Targeted indications	Gen Surg, Gyn, Peds	Natural Orifice Surgery	Multi-Specialty	Gen Surg Urology	Natural Orifice Surgery	Gen Surg, Gyn, Urology	Gen Surg	urology, gyn, gen surg, colorectal	Gen Surg, Gyn,	Gen Surg,
Level of autonomy	Assistive Minimal	Assistive Minimal	Assistive Minimal	Assistive Minimal	Assistive Minimal	Assistive Minimal	Manual	Assistive Minimal	Assistive Minimal	Assistive Minimal
Human robot interaction	Teleoperated	Teleoperated	Teleoperated	Teleoperated	Teleoperated	Teleoperated	Direct	Teleoperated	Teleoperated	Teleoperated
Architecture	Boom mounted Multi-arm	Endoluminal Flexible Double Arm	Multi-arm Bed based	2-arm/2 carts	Endoluminal Flexible Double Arm	Modular Multi-arm	2-arm/1 cart	Single port w 3 arms	single port Double arm	single port Double arm
Surgeon console	Open	Open	Open	None	Open	Open	None	Open	Open	Open
Instruments	Reusable 3 & 5 mm	Two 6 mm Reusable	Not available	None Camera Holder and Magnetic Retraction arm	Disposable	Semi Disposable (reusability yet to determine)	Uses standard laparoscopic camera and instruments	Multi-articulating reusable wristed instruments	disposable or reusable instruments	compact design that can be sterile processed in its entirety
Sustainability	High	High	Not available	High	Low	Low	High	High	Not available	High
US Regulatory approval	FDA: Gen Surg, Gyn, Peds	Awaiting Clearance	Under development	FDA: Gen Surg Urology	FDA: transoral; colorectal; general surgery; gyn; thoracic;	FDA Clearance in process	FDA: Gen Surg	Awaiting Clearance	Awaiting Clearance	Awaiting Clearance
Current market	> 50 Systems with > 10,000 Procedures	NA	NA	USA	USA	Canada, Italy, Chile, India Panama	USA, France	USA, Canada	USA	USA
Ideal stage	Stage 3	Stage 0	Stage 0	Stage 0	Stage 1	Stage 2b	Stage 2a	Stage 0	Stage 0	Stage 1

**Table 2:** Upcoming European abdominal surgical robotic systems

	Avatera	Versius	Dexter	Revo-i	Anovo Hominis	S Bitrack system
Manufacturer	Avatera Medical	Cambridge Medical Robotics CMR Surgical	Distal Motion	Meerecompany Inc	Momentis Surgical -	Rob Surgical Systems
Platform	Avatera	Versius	Dexter	Revo-i	Anovo Hominis	S Bitrack System
Country of origin	Germany— Jena	UK	Switzerland—Lausanne,	South Korea	Israel	Spain
Targeted indications	Urology, Gyn	Gen Surg, Gyn, Thoracic, Urology	Gen Surg, Gyn, Colorectal, Urology	Gen Surg, ENT, Gyn, Urology	Gynecology	Gen Surg, Gyn, Urology
Level of autonomy	Assistive Minimal	Assistive Minimal	Assistive Minimal	Assistive Minimal	Assistive Minimal	Assistive Minimal
Human robot interaction	Teleoperated	Teleoperated	Teleoperated	Teleoperated	Teleoperated	Teleoperated
Architecture	Multi-arm 3–4	4 independent cart-mounted arms	3 independent cart-mounted arms	Multi-arm	Transvaginal insertion of instruments	Multi-arm 3–4
Surgeon console	Open	Open with 3-d glasses	Open	Closed	None	Open
Instruments	Disposable 5 mm single use	Reusable 5 mm Instruments	Disposable 5 mm single use	Reusable with haptic feedback	miniature humanoid-shaped arms	Disposable 8 mm single use
Sustainability	Low	High	Low	High	Low	Low
Regulatory approval	Europe	Europe, Middle East, Asia, Australia, and Latin America	UK Europe	Korea	FDA- Gyn	Awaiting clearance
Current market	Europe	10,000 procedures (Mar 2023)	Europe	Korea, Russia, Kazakhstan, and Uzbekistan	USA	Europe
Ideal stage	Stage 1	Stage 2b	Stage 2a	Stage 2b	Stage 0	Stage 0

**Table 3:** Upcoming Asian abdominal surgical robotic systems

	KangDuo	Toumai	Hinotori	Shurui	Carina	Mantra	Micro Hand S
Manufacturer	Harbin Sagebot	Microport Medbot	Medicaroid	Beijing Surgerii Robotics Company Ltd	Ronovo Surgical	SS Innovation	Wego
Platform	KangDuo	Toumai	Hintori	Shurui	Carina	Mantra	Micro Hand S
Country of Origin	China	China	Japan	China	China	India	China
Targeted Indications	Urology, General Surgery	Gen Surg, Gyn, Thoracic, Urology	Gen Surg, Gyn, Urology	Urology, Gynecology	Gen Surg, Gyn, Thoracic, Urology	abdomen, pelvis, thorax and cardiac	Gen Surg, Colorectal, HPB
Level of Autonomy	Assistive Minimal	Assistive Minimal	Assistive Minimal	Assistive Minimal	Assistive Minimal	Assistive Minimal	Assistive Minimal
Human Robot Interaction	Teleoperated	Teleoperated	Teleoperated Demonstrated long range	Teleoperated	Teleoperated	Teleoperated	Teleoperated
Architecture	Multi-arm 3–5	4 independent cart-mounted arms	Multi-arm	4—Arm with Single port	4 independent cart-mounted arms	5 Modular independent cart-mounted arms	4 Arm
Surgeon Console	Open	Closed	Closed	Semi closed	Closed	Open	Open
Instruments	Reusable 8 mm (ten uses)	Reusable instruments with force feedback	Reusable	Reusable	8 mm wristed (single-use) & 5 mm non-wristed (reusable)	More than thirty 8 mm reusable instruments	Reusable
Sustainability	High	High	High	Low	Low	High	High
Regulatory Approval	China	China Europe	Japan	China	Awaiting clearance	India, Europe Awaiting FDA clearance	China
Current Market	China	China Europe Africa	Japan	China	China	India, Middle East, Latin America, Europe	China
Ideal stage	Stage 2b	Stage 2b	Stage 2b	Stage 1	Stage 0	Stage 1	Stage 2b

### Asensus surgical—Senhance & Luna

**Fig. 1:**
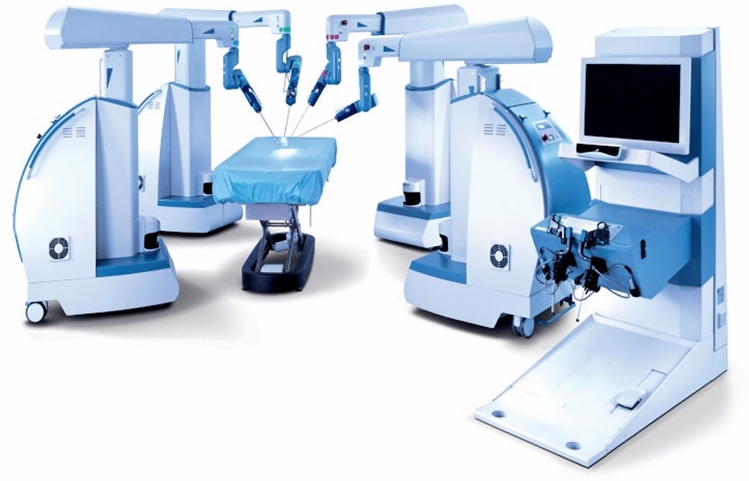
Asensus Senhance platform

Asensus, previously called Transenterix is based in Durham, North Carolina, USA. The company acquired an Italian robot named ALF-X and re-branded it as “Senhance.” This robot (Fig. 1) is a multi-arm system currently approved for bariatrics, colorectal, general surgery, gynecology (benign), and Pediatrics in the US. It is also approved for Urology outside the US. The system is modular with multiple independent arm carts (3 or 4 indications vary by market). The arms are connected to towers in a boom-mounted design where the operative arm is coming from the top. In Europe, the system has a CE mark for four independent arms while in the US the FDA approved only three arms. The console is open with 3D passive glasses; it is equipped with eye tracking to control the camera, haptic feedback, and a pedal to activate or freeze the system. The controllers are designed to resemble laparoscopic instrument handles. The software allows zooming, panning, and measuring inside the abdominal cavity in a straight line or contour mode that follows the anatomy profile. It can tag anatomical structures, and those digital tags will move following the anatomy. It uses reusable 3 mm and 5 mm straight instruments and 5 mm instruments with seven degrees of freedom. For pediatric patients, the company believes its 3 mm instruments offer a unique advantage [15]. All instruments are reusable with some single-use offerings. The system can work with any laparoscopic trocar and self-acquires the fulcrum during the docking process without attaching the arm to the trocar directly. There is a wide array of instruments, including ultrasonic dissector. The system can accommodate a large variety of laparoscopic cameras, either 2D or 3D. It is compatible with any energy source. The system has been in clinical use since 2016 and there is clinical data on over 800 cases demonstrating safety in hernia repairs cholecystectomies, and prostatectomies [16]. Data on bariatric surgery [17] and pediatric surgery [18] procedures is emerging.

**Fig. 2:**
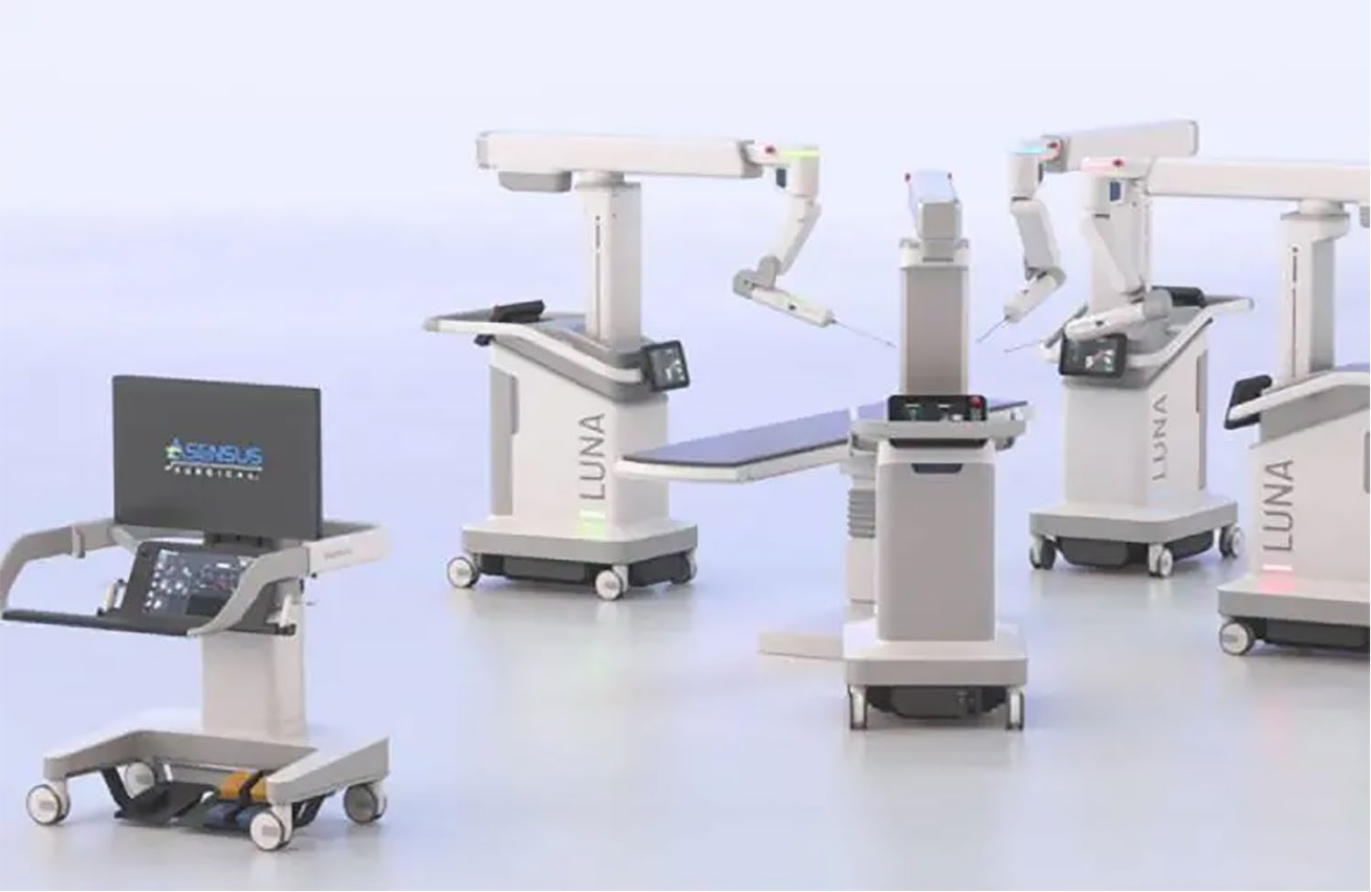
Asensus new Luna platform

Asensus’ newer Luna system (Fig. 2) aims to build upon the Senhance platform, and the company locked down the system’s design in early 2024, with 510(k) applications to be submitted to the FDA by the end of 2024 and a commercial pilot launch in the latter half of 2025 [19].

Asensus Medical has entered exclusive negotiations for acquisition by Karl Storz as of April 2024.

### Avatera medical—Avatera

**Fig. 3:**
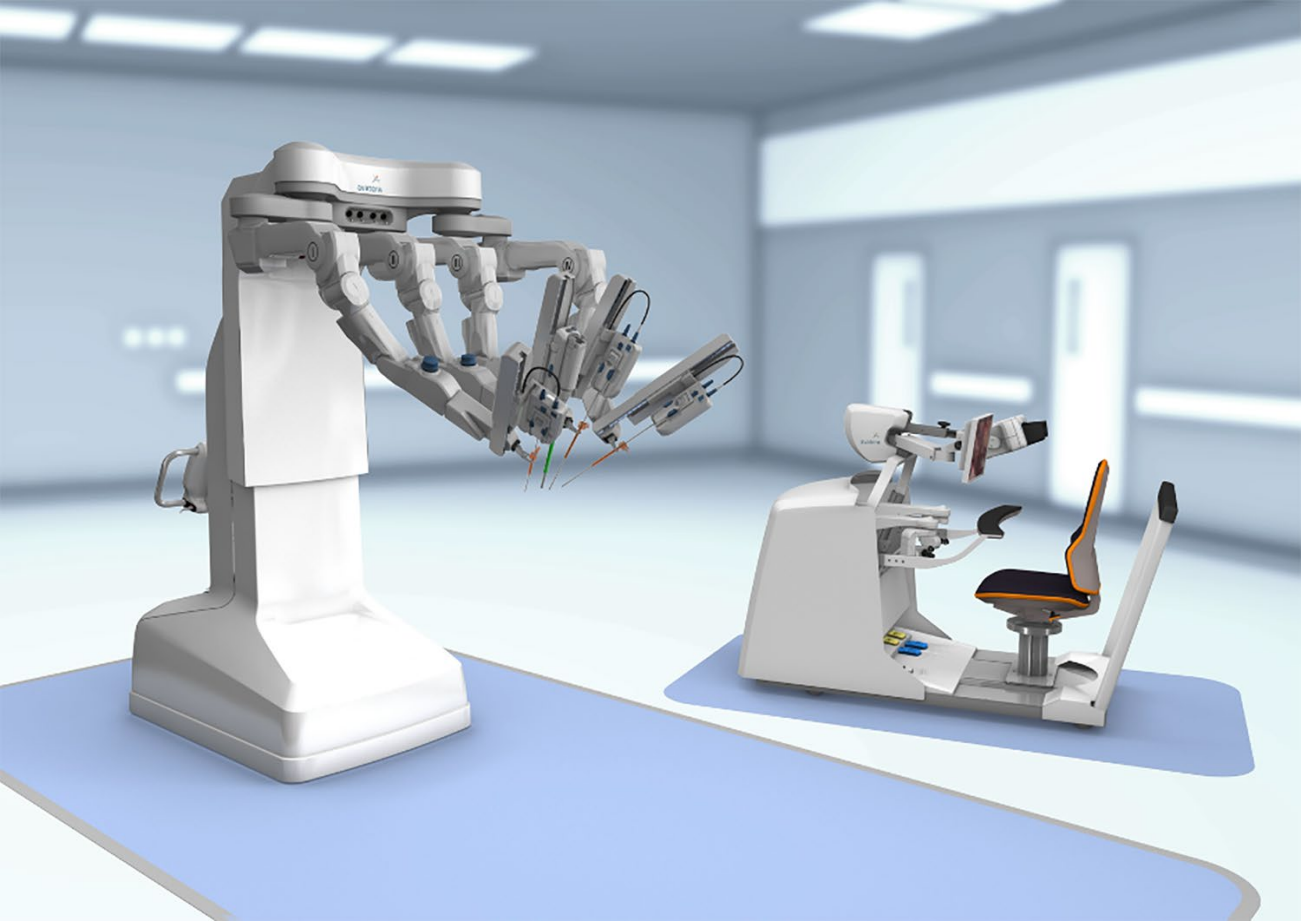
Avetera platform

The Avatera (Avateramedical, Jena, Germany) was founded in 2011 and represents the first German robotic surgical system (Fig. 3). It is a 2-component robotic system consisting of a robotic cart with mounted 4 robotic arms and a closed surgeon console [20]. The console has a slender eyepiece that does not obstruct the surgeon’s mouth or ears, an integrated and flexible seat, and both haptic, manual input devices and footswitches. It possesses a full HD resolution camera with QXGA resolution screen, and the image appears the same size as natural field of view. 5 mm disposable, single use instruments with a 7 degrees of freedom range of movements. Instruments include Metzenbaum scissors (bipolar), Atraumatic grasper, Maryland dissector (bipolar) and Needle holder. Use of fully disposable instruments working exclusively with bipolar energy is a unique feature of this platform with the company marketing this as avoiding the risk of cross contamination and decreasing sterilization costs. It completed its first ten surgeries to remove prostate and kidney tumors in May 2022 [21] and is approved in Europe with CE Mark approval for urology and gynecology. It is awaiting FDA approval.

### Cambridge medical robotics (CMR)—Versius

**Fig. 4:**
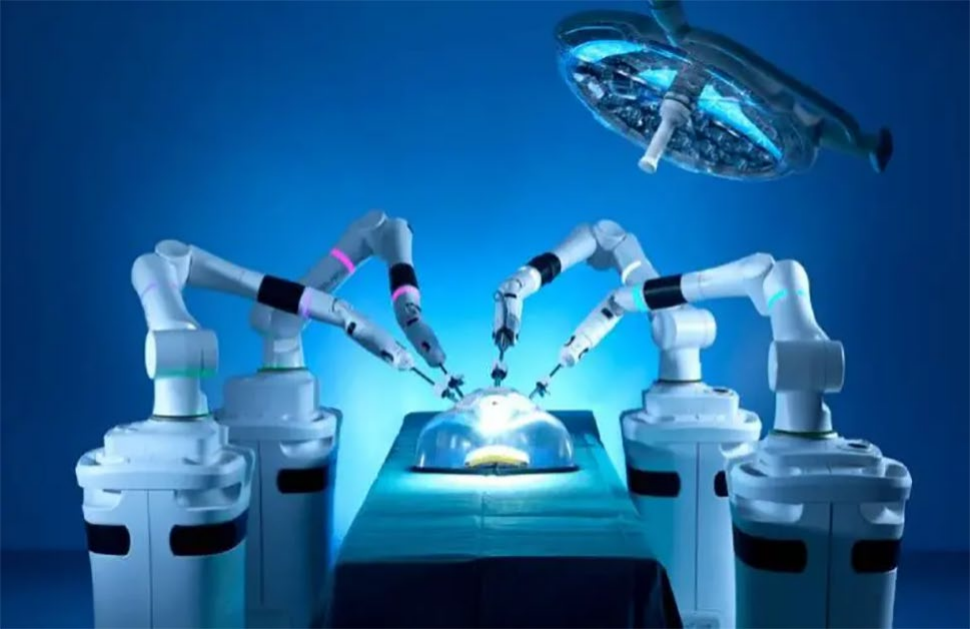
CMR Versius platform

**Fig. 5:**
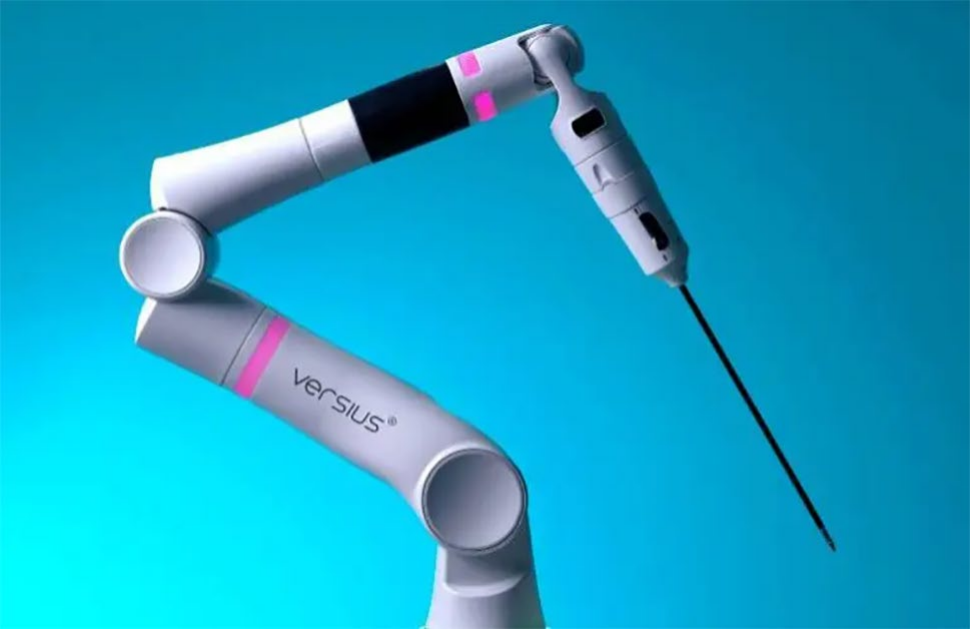
CMR Versius arm with 360° rotation

CMR is based in Cambridge, United Kingdom, and their Versius System (Fig. 4) is currently approved for general surgery, gynecology, thoracic surgery, and urology mostly in Europe under the CE mark as well as in Asia Pacific. It is awaiting FDA approval. More than 15,000 procedures have been performed using the system as of February 2024 [22]. The modular system has 4 independent cart-mounted arms with 360° wrist movements (Fig. 5). The arms have a feature that allows repositioning of the arm segment without removing the instruments. The reduced size of the carts makes it more portable and facilitates moving the system between rooms or in dedicated storage [23]. The Surgeon console is an open design with 3D passive glasses that allows both sitting or standing. The controllers are designed to resemble laparoscopic instruments. The system is completely controlled with the handles and no available pedals. The console is also equipped with a video recording device. It uses 5 mm Instruments of variable lengths with seven degrees of freedom. Monopolar and bipolar energy are available.

### Distal motion—Dexter

**Fig. 6:**
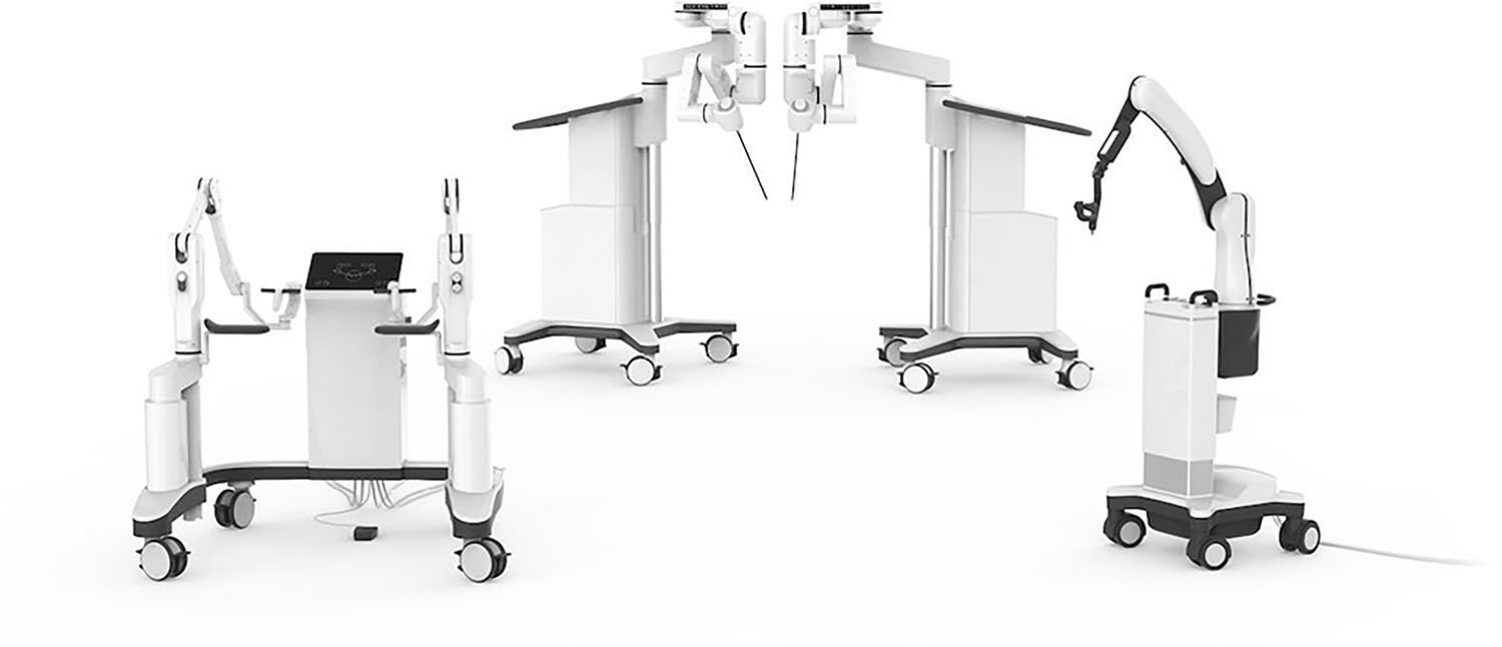
Distal Motion—Dexter Platform

The Dexter Robotic System (Fig. 6), created by Distalmotion, is a “hybrid” surgical platform providing surgeons the ability to change between robotic and laparoscopic approaches quickly from the sterile console. The sterile console is open, allowing the surgeon to sit or stand, with two handle grips, a clutching foot pedal, and a camera foot pedal. It consists of two moderate- to low-profile robotic arms, each mounted to a separate cart, and one endoscopic arm that can be mounted to either the endoscopic cart or the operating table. On each arm, the boom can be adjusted vertically and horizontally to achieve the desired height and position of the arm while leaving sufficient working space at the bedside. The endoscopic arm is compatible with any 5-10 mm 3D laparoscopic imaging system, allowing surgeons to select which 3D/fluorescence imaging system they prefer. The system functions with any 10–12 mm standard laparoscopic trocars and utilizes fully wristed, single-use instruments, which negate the need for reprocessing and associated concern for deterioration due to repeated use. The company’s 8.3 mm instrument panel includes a needle holder, monopolar hook, monopolar scissors, bipolar Maryland dissector, and bipolar Johann grasper which have 7 degrees of freedom and a 75° range of angulation movement. Arms are “docked” by use of a tracking device that is temporarily placed in the laparoscopic trocar for positioning of the arm, which moves freely without attachment to the trocar itself. The LAP button on each instrument arm allows for quick transition between robotic and laparoscopic approaches by folding the robotic arm away from the surgical field without undocking [24].

The platform received European CE Mark in December 2020 for use in general, urological, and gynecological surgery and has been used in clinical practices across Europe since 2021. The Dexter platform is currently pending FDA approval for US usage. The company promotes Dexter’s compatibility with all existing imaging systems and energy generators along with their tailored, cost-effective financing packages to make access to robotic surgery more economical [25]. A published study reporting on the first 12 cases utilizing Dexter included 2 robotic assisted ventral mesh rectopexies, 8 oncologic right colectomies, 1 transverse colectomy, and 1 ileocecal resection. In the 8 oncologic colectomies, central vascular ligation was performed using the robotic platform and medialization and transection of the colon was carried out laparoscopically. Given the surgeon’s ability to remain scrubbed in and the use of the LAP button, the transition time from laparoscopy back to the robotic platform was 15–30 s. There were no reported platform-related adverse events intraoperatively [26].

### Endoquest robotics—Endoluminal Surgical (ELS)

**Fig. 7:**
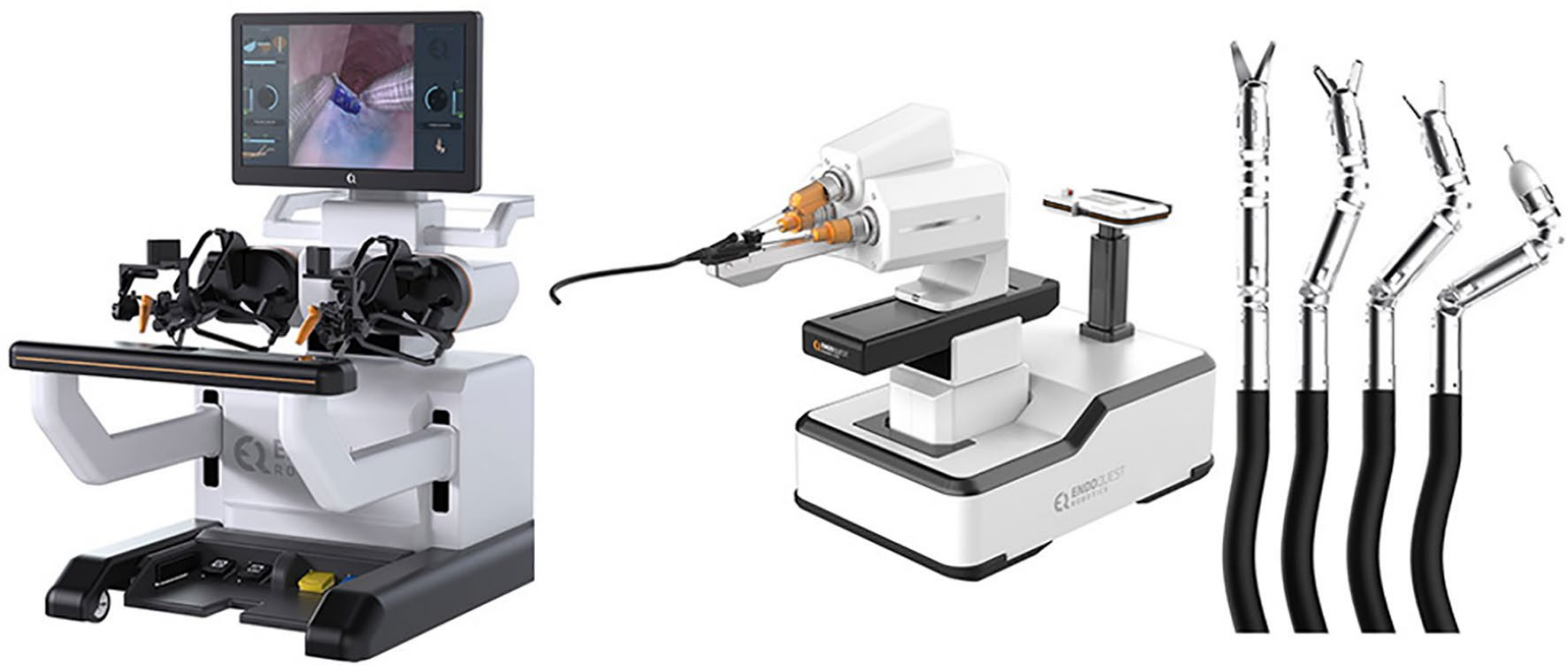
EndoQuest ELS Platform

ColubrisMX was the world’s first endoluminal robotic surgical system allowing upper and lower gastrointestinal surgery through a trans-oral or trans-anal approach. The company rebranded as Endoquest Robotics in 2022 and their system is called Endoluminal Surgical (Fig. 7). Initially designed towards NOTES procedures, the company also lists transumbilical access as an application for their platform. The platform consists of an open console and a single patient cart. The effectors consist of a flexible 3.7 mm camera and two 6 mm flexible instruments with 7 degrees of freedom, all housed in a flexible shaft. No human cases have been reported to our knowledge, and no FDA clearance date has been announced.

### Johnson & Johnson—Ottava

**Fig. 8:**
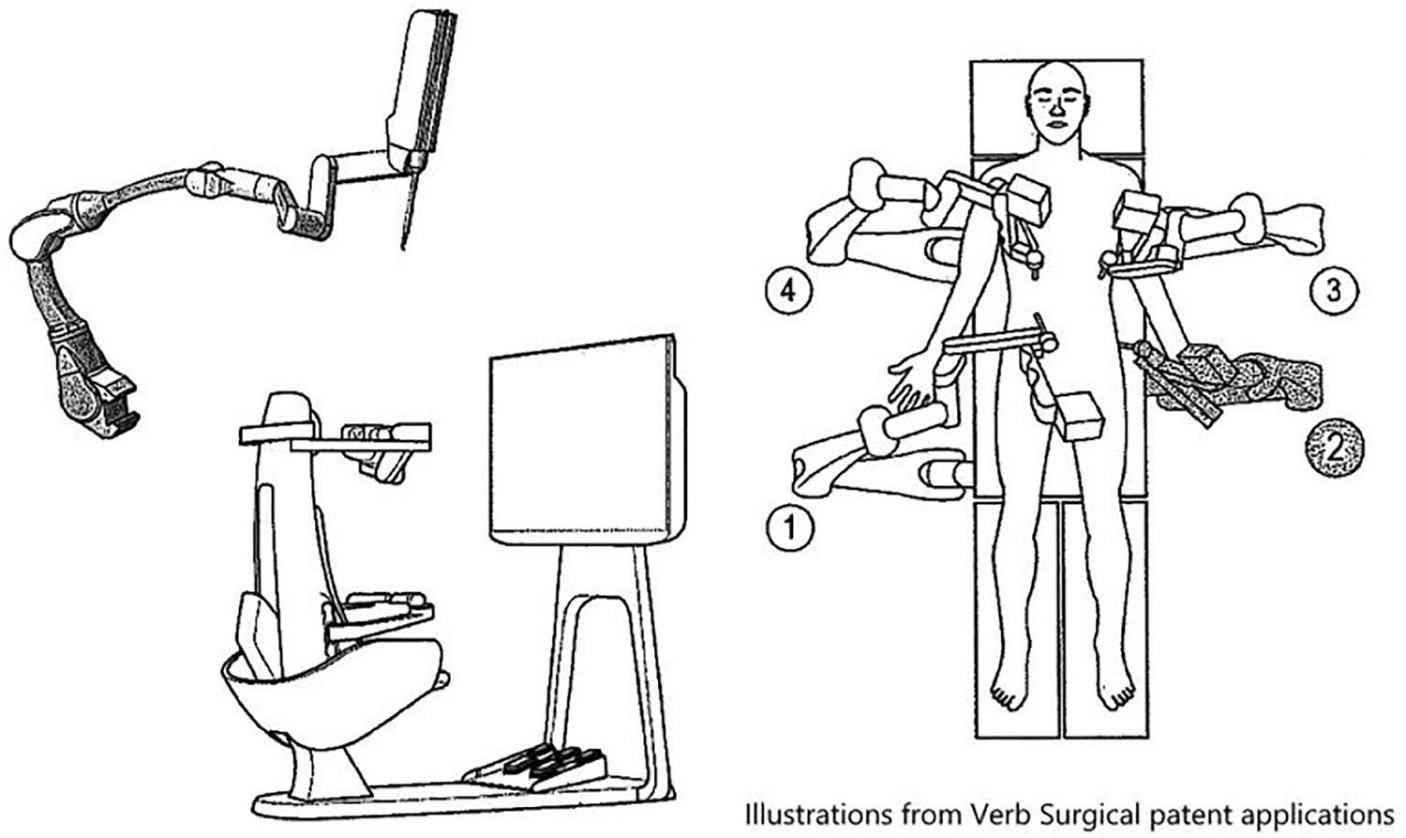
Ottava patent application images

Auris and Verb Surgical, who had previously been independently working on developing robotic platforms, were acquired by Johnson and Johnson in the last 5 years. This has resulted in one of the most anticipated platforms in the United States although very few details are publicly available. OTTAVA incorporates four robotic arms into a standard size surgical table (Fig. 8). This unified architecture allows for an invisible design, with the robotic arms available when needed or stowed under the surgical table when not. The design removes barriers to movement and collaboration in robotic operating rooms and offers surgical teams the freedom and flexibility to adapt to clinical workflows and individualized patient needs. The system’s “twin motion” feature—unified movement of the table and the robotic arms—is designed to allow surgical teams to address important clinical needs during surgery, such as the ability to reposition a patient without interrupting the procedure. The robotic team has been working with Ethicon franchise in France to bring custom engineered Ethicon instruments to Ottava. J&J is targeting the second half of 2024 for filing an IDE to the FDA to allow clinical trials in the US.

### Levita magnetics—MARS

**Fig. 9:**
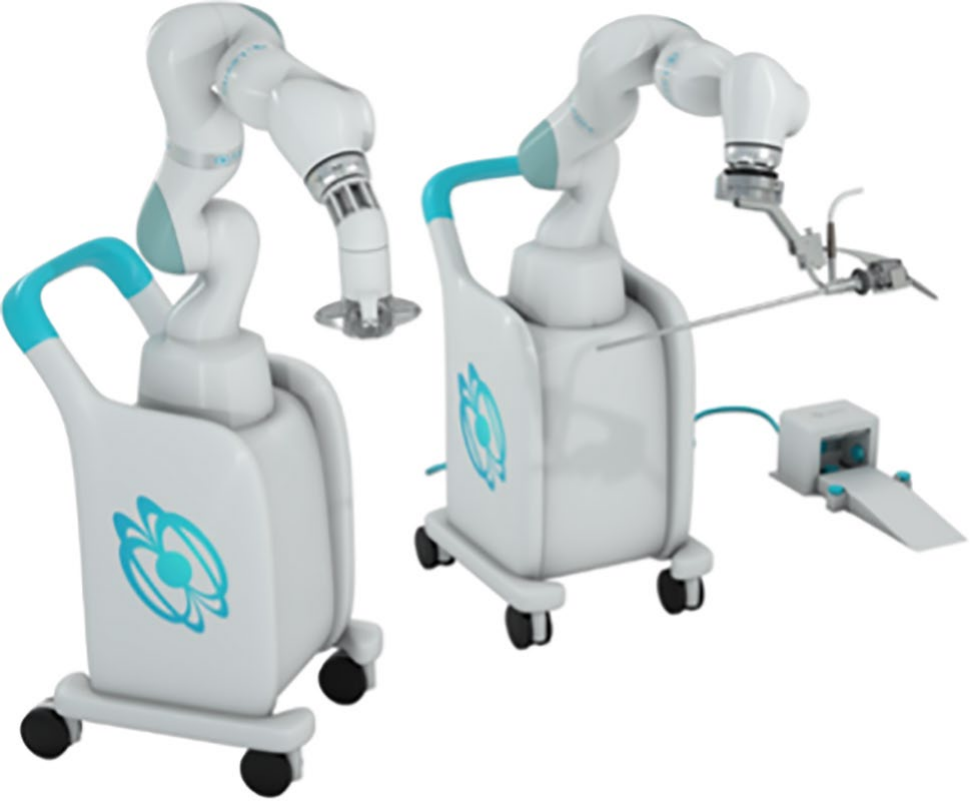
Mars Surgical Platform

**Fig. 10:**
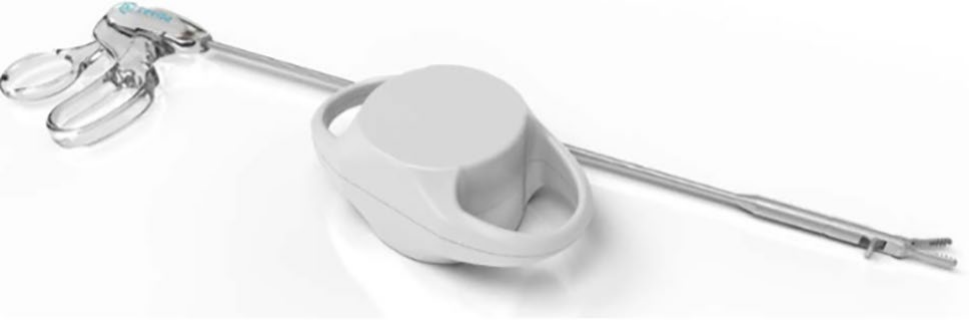
Mars Magnetic grasper and controller

The MARS platform is designed to be a “surgical assistant” platform. It is composed of two arms on two separate carts, one arm being a camera holder and the second is a magnetic retraction arm (Fig. 9). It is an open (no console) platform, aimed to be used with a conventional laparoscopic tower. Both arms are controlled with a foot pedal system. The first arm is a camera holder, latched on to any laparoscopic camera. The second robotic arm has a powerful magnet (magnet actuator) that remains outside the body on the surface of the abdomen (Fig. 10). It can attract and control an internal magnet placed in the abdomen through a standard 5 mm port. This internal magnet is a deployable grasper attached to a standard 5 mm instrument, the target (ex. gallbladder) is grasped in a traditional laparoscopic fashion and the tip of the grasper can then be released. It will then be retracted by approaching the magnetic arm on the other side of the abdominal wall. This allows to control retraction through the abdominal wall without the constraint of a port. The force of retraction is controlled by placing the external magnet closer or further from the abdominal wall.

The platform has obtained FDA clearance in August 2023 for the following indications: Gallbladder, Bariatric, Colorectal and Prostate procedures. To date the company reports that about 180 cases have been performed (as of 2/2024) in the US and Chile (unpublished). The first 30 cases were published in 2022: 15 gastric sleeves, 14 cholecystectomies, and 1 Roux-en-Y gastric bypass. Showed ability to reduce one port in all 30, no serious adverse events and 2 minor device-related adverse events.

### Medicaroid—Hinotori

**Fig. 11:**
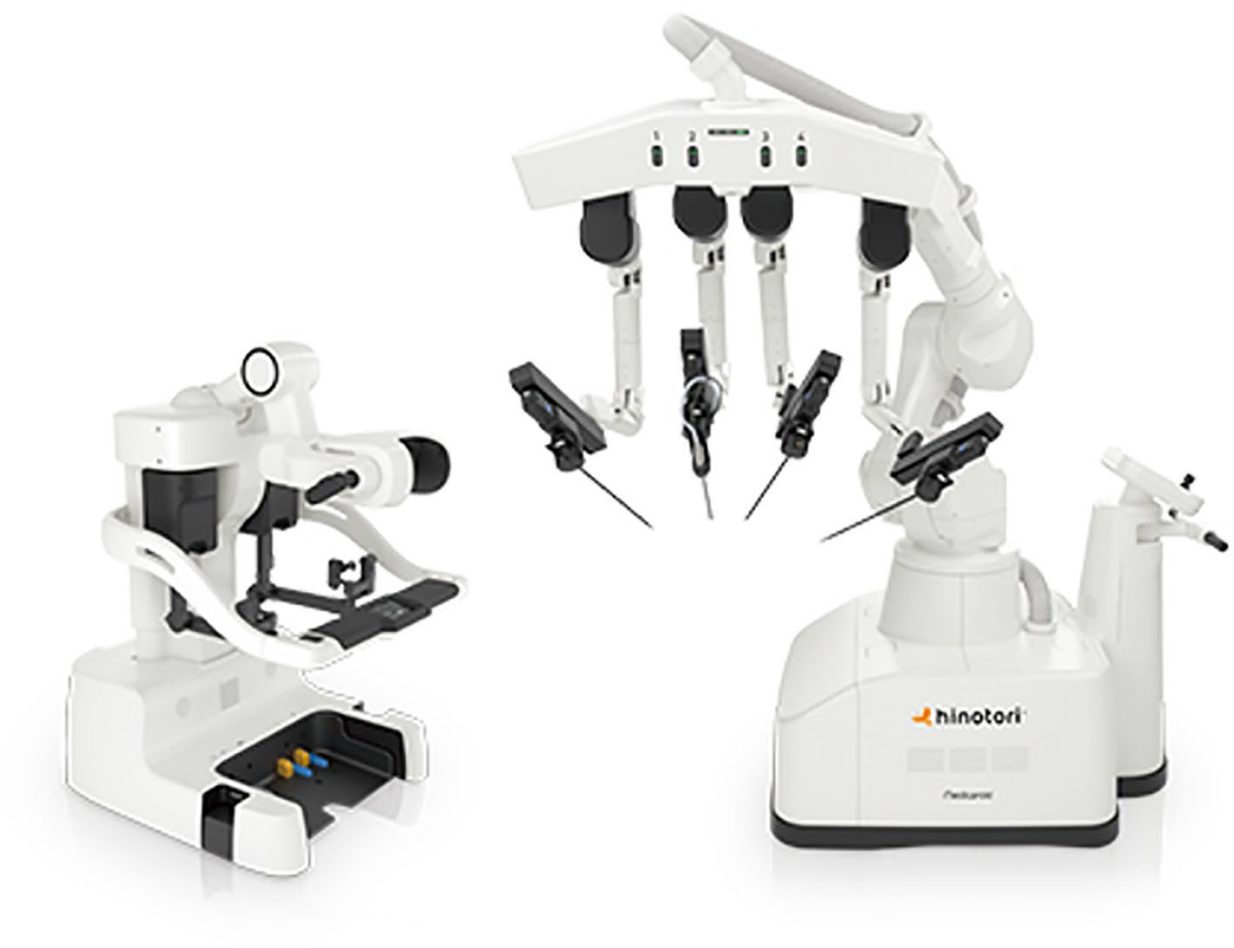
Medicaroid Hinotori System

Medicaroid’s robotic system, Hinotori (Fig. 11), is currently used in Japan for gynecologic, urologic, and general surgery procedures. The system received regulatory approval from the Japanese Ministry of Health, Labor, and Welfare (MHLW) for use in urological procedures in August 2020 and was granted expanded approval to gynecological and general surgeries October 2022. Medicaroid applied for additional approval for use in thoracic surgery in late 2023. The system consists of a closed surgeon cockpit, a four-arm operating unit, and a vision unit. The ergonomically designed surgeon cockpit can be adjusted to fit the surgeon’s preferred posture with the option to rotate the 3D endoscope viewer which contains a high-definition screen. Surgeons operate the camera and instruments using two hand controls and foot switches. Emphasis is placed on the operating unit’s ability to perform sophisticated movements using the eight-axis operating arms and compact design with arms arranged in a linear fashion. The linear design and movement capability of the arms is aimed to reduce arm-to-arm and arm-to-assistant contact. Unit software sets the pivot point of the reusable instruments and allows instruments to be operated without arm-to-trocar docking. There is limited information regarding the instruments Medicaroid created for use with this platform, though they are reusable up to 10 times through 8 mm ports [27].

The company promotes its mission of improving access to healthcare in Japan through remote robotic-assisted surgery. In October 2023, the Hinotori platform was used to complete a remote surgery demonstration with the surgeon cockpit located in Singapore and the operating unit in Nagoya, Japan, approximately 5000 km apart [28].

### Medrobotics Corp—Flex® Robotic System

**Fig. 12:**
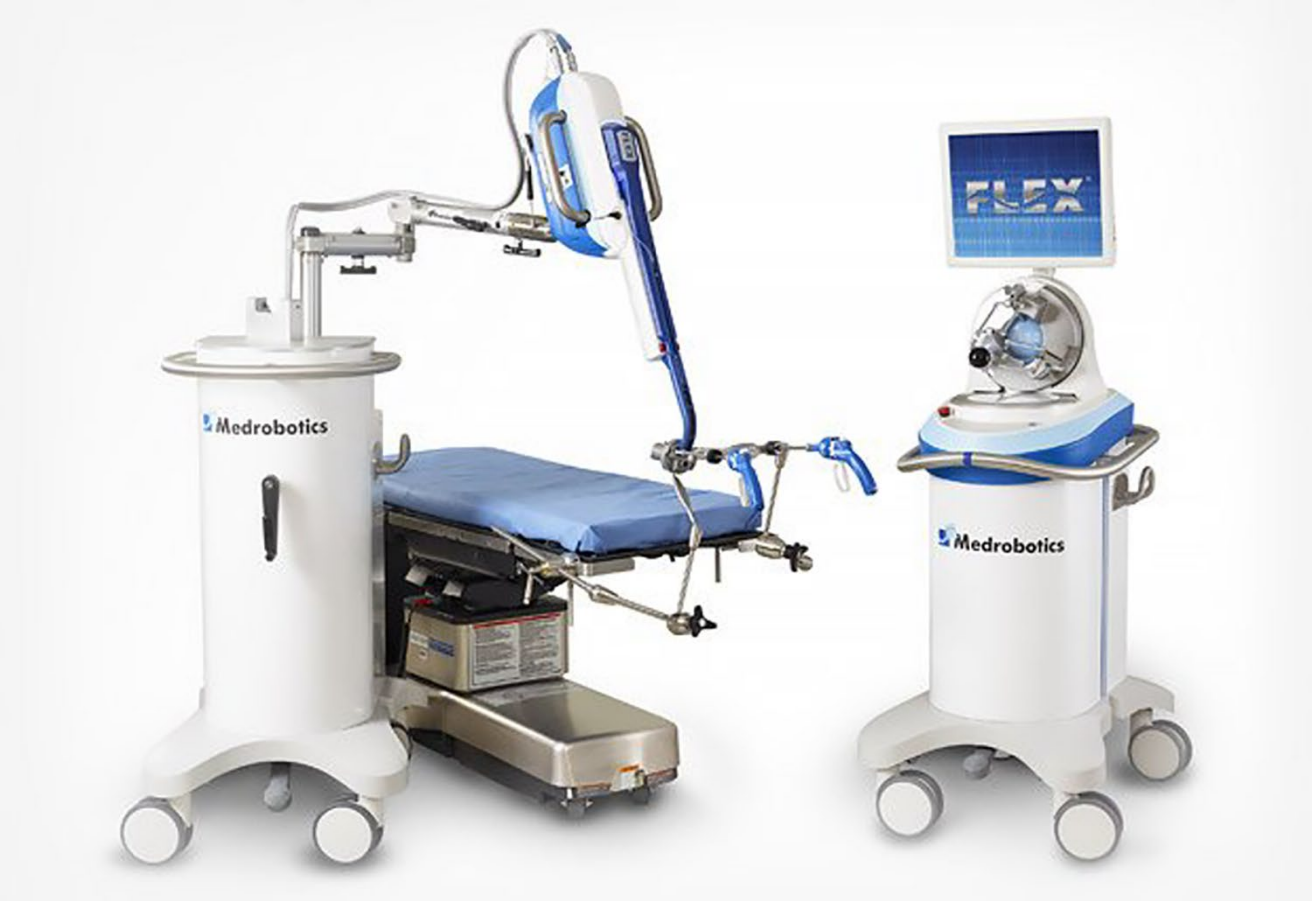
Medrobotics Corp—Flex Robotic System

Flex Robotic System created by Medrobotics Corp was a flexible robotic endoscope (Fig. 12) used for obtaining surgical site access to the oropharynx, hypopharynx, and larynx and subsequently transanal. The system was granted FDA approval for use in transoral applications in 2015, and colorectal applications in 2017. Additionally, it received a CE mark and obtained Australian TGA approval for use in colorectal procedures. The Flex system consisted of an open console featuring a three-dimensional, high-definition screen with an operator-controlled joystick and a base that attaches directly to the disposable flex drive scope. The system functioned through a single access point and the 170 cm flexible, multi-linked scope allows for access to difficult surgical sites. The endoscope contained two concentrically organized segments which allow steering like traditional endoscopic technology [29]. Once positioned at the target site, the scope became rigid to create a sturdy operating platform to allow for usage of flexible surgical instruments. The ability of the scope to move along non-linear, circuitous paths provided surgeons 180° flexibility around anatomy to perform procedures. The flex drive scope featured external electrical connections for attachment of cameras and LEDs and had a lumen which allowed for tubing containing camera lens washer and additional accessory pathways on both sides of the camera for passage of surgical instruments. Flex featured an open architecture allowing for use of third party surgical and interventional instruments. Medrobotics offered a full suite of flexible 3 mm articulating surgical instruments including monopolar scissors, monopolar spatula, laser holder, needle driver, monopolar needle knife, fenestrated grasper, and monopolar Maryland dissector [30].

Medrobotics filed for bankruptcy in early 2022 [31].

### Medtronic—Hugo

**Fig. 13:**
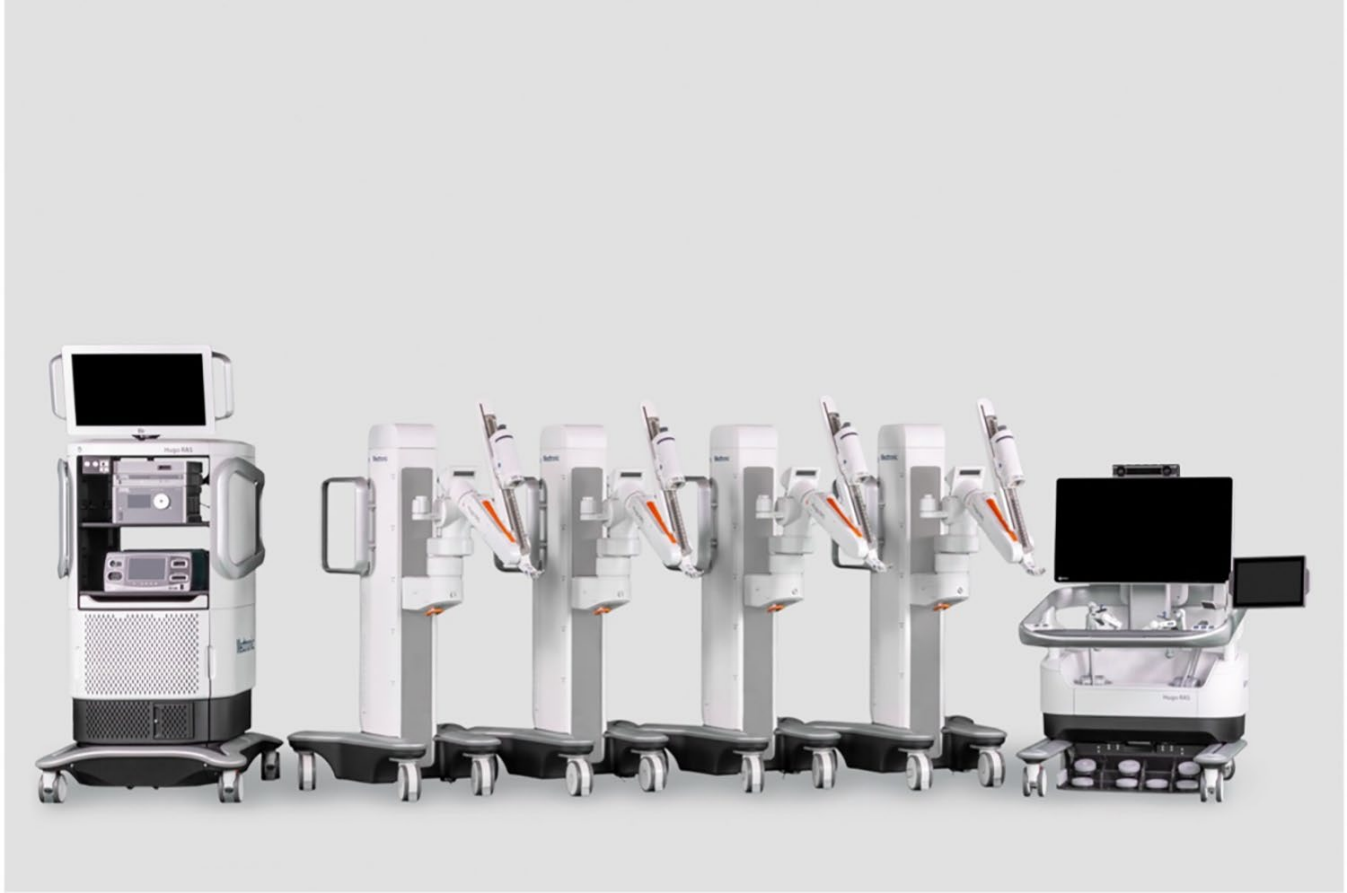
Medtronic—Hugo RAS System

Hugo Robotic Assisted Surgery platform was launched by Medtronic in 2021. The system has obtained CE mark for gynecological, urological, and general surgery procedures in Europe, Canada, Japan, and Taiwan. It is awaiting FDA approval for use in the US. The Hugo system (Fig. 13) features an open console and the option for use of up to four modular arm cart units. The open console features a 32-inch 2D or 3D display with pistol-grip hand controls and foot pedals. 3D visualization requires the operator to wear non-polarized eyewear. The unique pistol-grip hand controls differ from other robotic platforms by resembling the grip of traditional laparoscopic tools. Additionally, the system features a ‘rotation multiplier’ and allows for up to 520° of instrument rotation. The platform does not offer near-infrared fluorescence imaging. Foot pedals are used to drive the camera and manage energy devices. Hugo utilizes an 11 mm 3D HD Image1-S endoscope which can be used with any of the arm carts. Each of the arm carts features a 6-jointed robotic arm with a laser alignment system used for docking the arm into position independent of the other arm carts. The system features versatile docking capabilities with the ability to adjust the arm tilt, height, and docking angle. One concern users have noted is the bulkier arms and long instrument tracks compared to other platforms, which could predispose the platform to collisions [32]. Hugo RAS utilizes wristed instruments featuring seven degrees of freedom which are semi-disposable. There is not yet data on the reusability of the instruments.

### Meerecompany—Revo-I

**Fig. 14:**
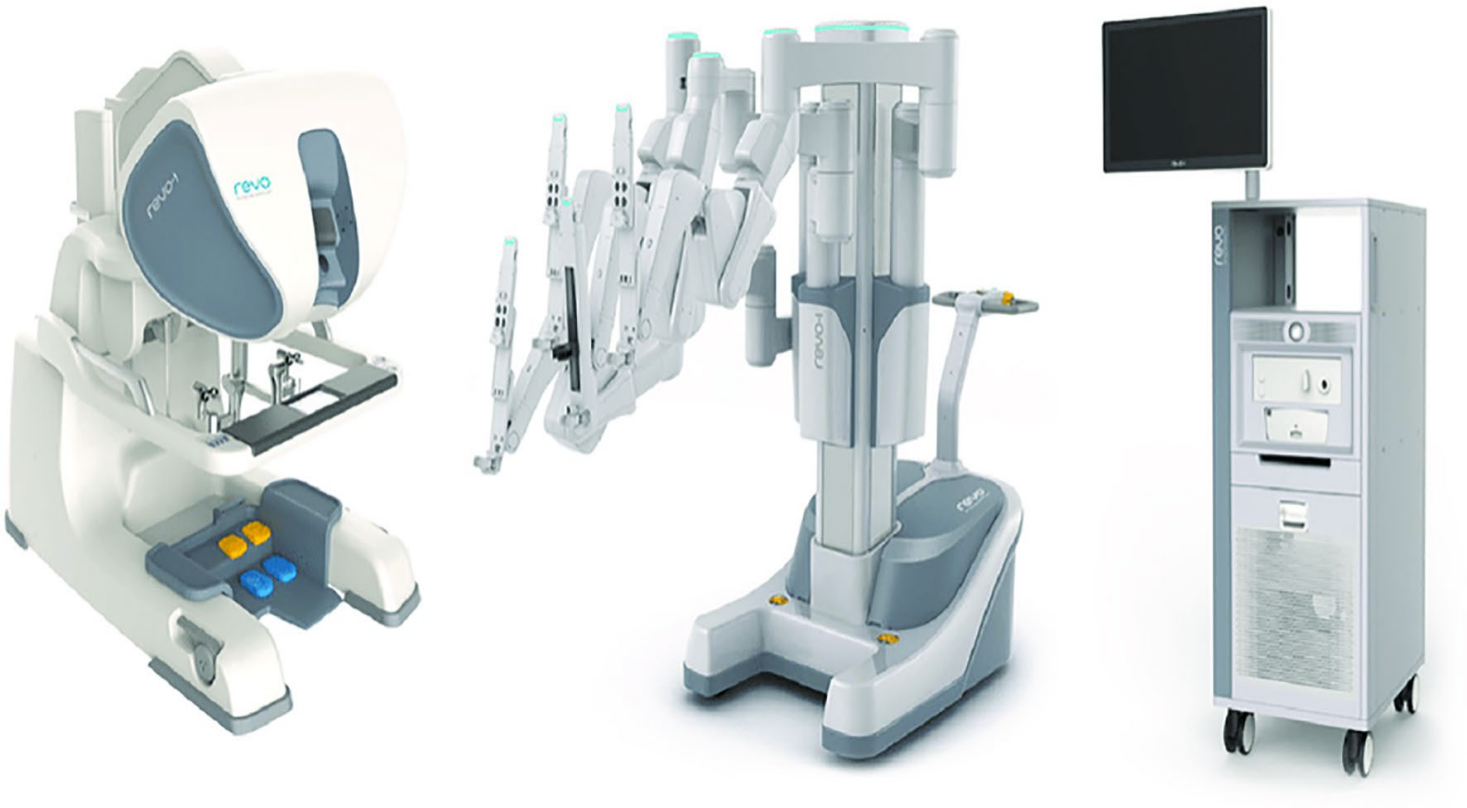
Meerecompany—Revo-I System

Revo-i (Fig. 14) by Meerecompany was the first Korean surgical robot. It has obtained regulatory approval locally (Ministry of Food and Drug Safety approval (MDFS, Korea) where it is currently used for urological, gynecological, ENT, and general surgery procedures. It is also used in Russia, Kazakhstan, and Uzbekistan. The platform features a closed surgeons console with ergonomic design features including adjustable headrest, armrest, and foot pedals. The foot pedals are used to control the Revo 10 mm 3D HD endoscope and electrosurgical energy. The operation cart features four multi-jointed robotic arms, three being utilized for operating and one camera arm. The system uses fully-wristed, reusable instruments which are compatible with commercial laparoscopic trocars. A unique feature of Revo-i is the option for using advanced ultrasonic energy in addition to standard monopolar and bipolar energy. The Revo sonic instrument utilizes ultrasound vibrations to simultaneously seal and cut [33]. The platform can also provide surgeons with “extensive force use” warning messages while operating the console [33]. Revo-i also features Revo-Sim VR, a virtual training console with guided training modules for operators to gain familiarity and practice with the platform.

### Momentis surgical—Anovo Hominis

**Fig. 15:**
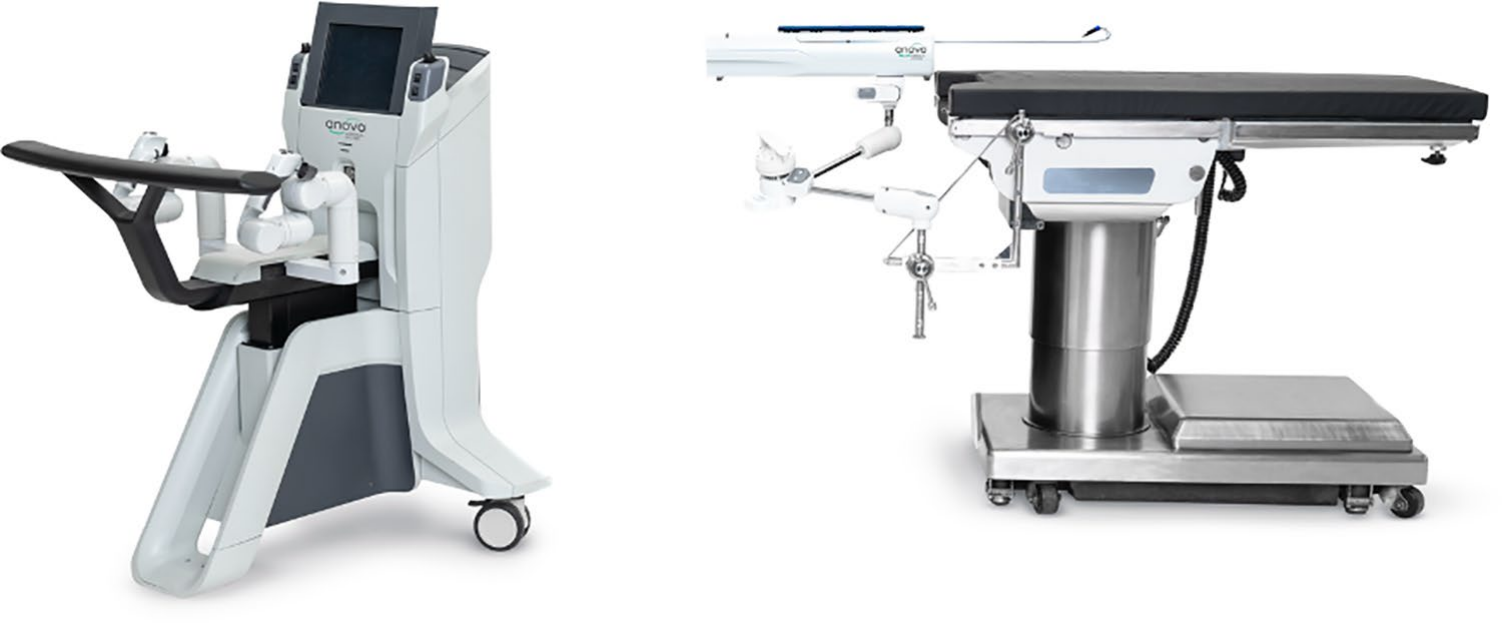
Momentis Surgical—Anovo Hominis Surgical System

**Fig. 16:**
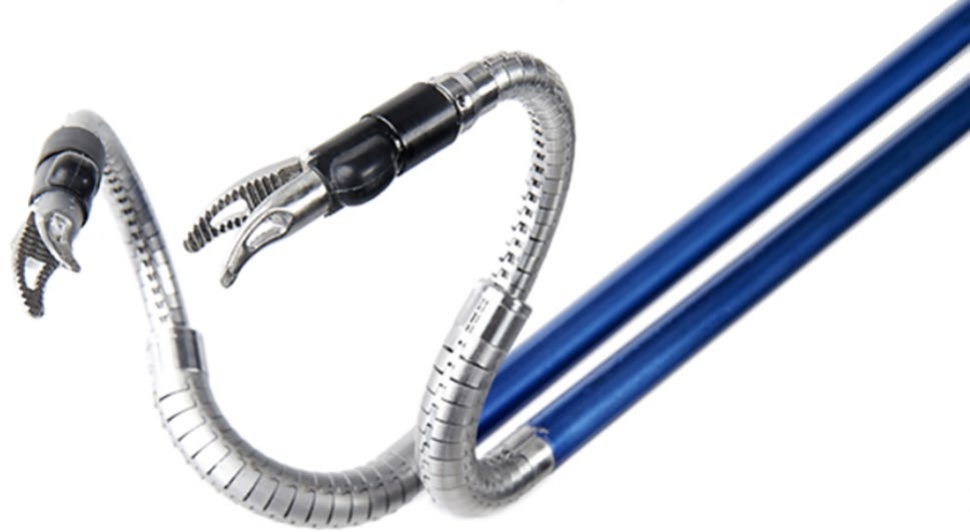
Momentis Surgical—Anovo Hominis Surgical Arms

The Anovo Hominis Surgical System was created by Momentis Surgical (formerly Memic) for use in gynecological procedures such as benign hysterectomy, salpingectomy, oophorectomy, adnexectomy, and ovarian cyst removals. It was granted FDA approval for these procedures in 2021. It is currently used in 3 hospital systems in Florida and 1 hospital in Texas. The system (Fig. 15) features a surgeon’s console and a robotic motor control unit which contains two miniature humanoid-shaped arms. Weighing only 13 pounds, the motor control unit mounts to the operating room table. The single-use arms contain shoulder, elbow, and wrist joints to perform precise movements (Fig. 16). The surgeon’s console is an open platform where each arm is controlled with a joystick and thumb stick. The thumb sticks are used for baseline retroflexion, insertion, and removal of the arms while the joysticks are used to control the end effectors. End effectors feature a spatula used for monopolar electrosurgical energy delivery and a fenestrated grasper which can apply bipolar electrosurgical energy. Electrosurgical energy is delivered through foot pedals on the surgeon’s console. For visualization, Anovo is compatible with third-party standard laparoscopes via a single transabdominal port and visual guidance system. Instruments are available through the reusable GYN trocar kit which allows for entry into the pelvic cavity through the pouch of Douglas [34].

### Moon surgical—Maestro

**Fig. 17:**
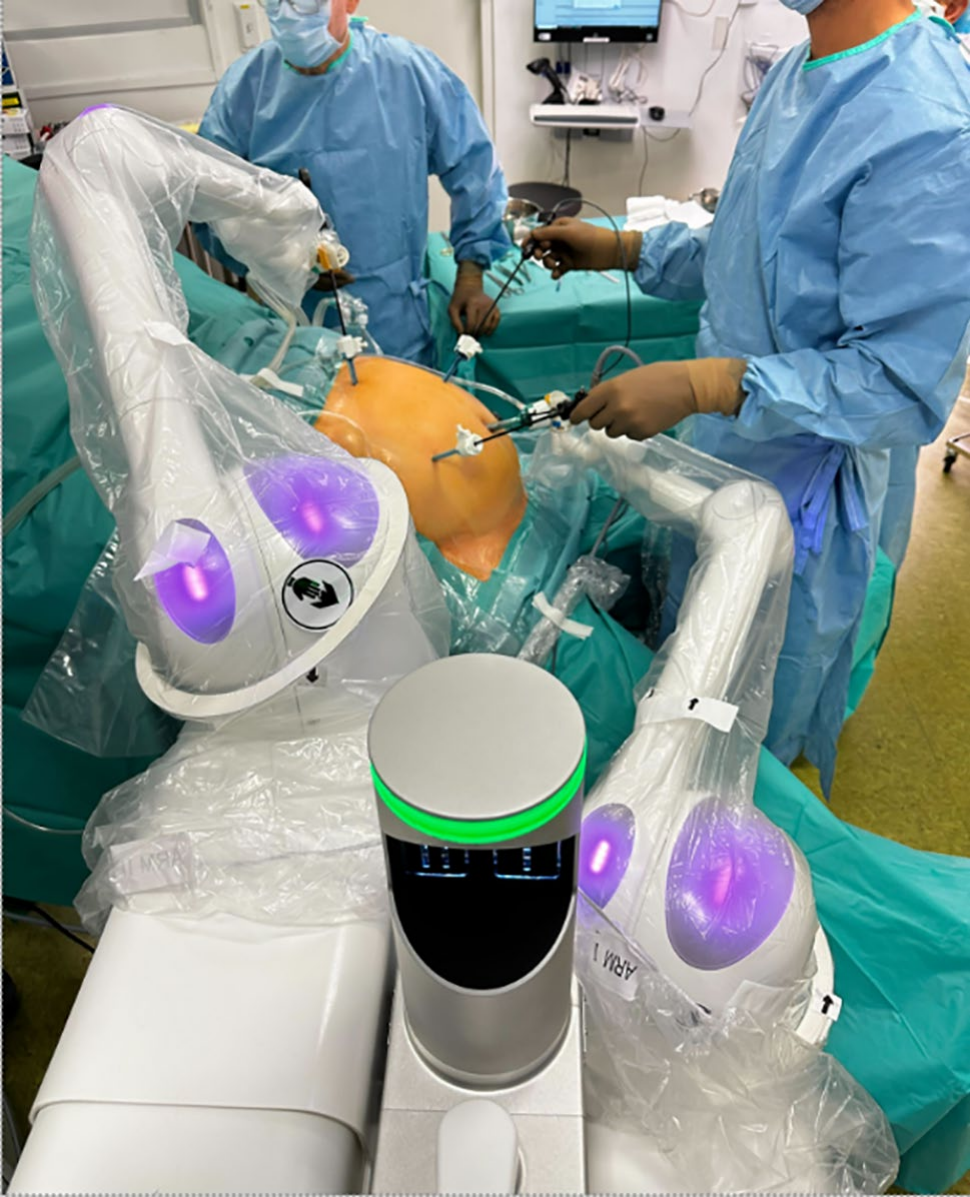
Maestro system from Moon Surgical

The Maestro Surgical platform is designed to be a “surgical assistant” platform. It is composed of two arms on a single cart, one arm being a camera holder and the second is an instrument holder (retraction) (Fig. 17). It is an open (no console) platform, aimed to be used with a conventional laparoscopic tower. The first arm is a camera holder, latched on to any laparoscopic camera, the second arm is latched to a standard 5 mm laparoscopic instrument such as a locking grasper. Both arms are controlled with a foot pedal system, however, in the most recent iteration (CE only at the time of writing) the imbedded AI is designed to allow the camera to track and follow instruments automatically. The initial version of the Maestro (pre-commercial) was cleared by the FDA in 2022. The commercial version of the system is expected to obtain FDA clearance in Q3 2024 and the AI automatic camera control in 2025. The price of the system has not been officially announced yet, but the company is planning on a monthly subscription model, based on usage. To date the company reports that about 190 cases have been performed (as of 2/2024), with the vast majority performed in EU (5 in the US). Cases performed include cholecystectomy, colorectal, foregut, bariatric, abdominal wall, and gynecological procedures. The first 10 cases—all cholecystectomies—were published in 2023, with no device-related complications [35].

### Rob surgical systems—S Bitrack System

**Fig. 18:**
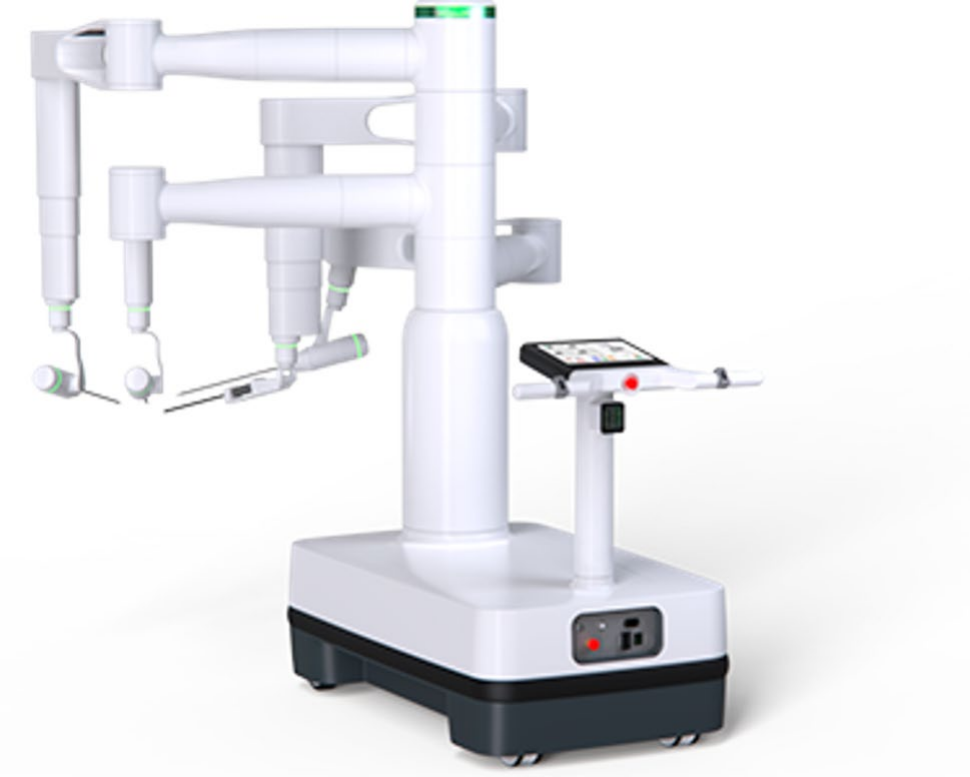
Rob Surgical Systems—S Bitrack System Operating Unit

**Fig. 19:**
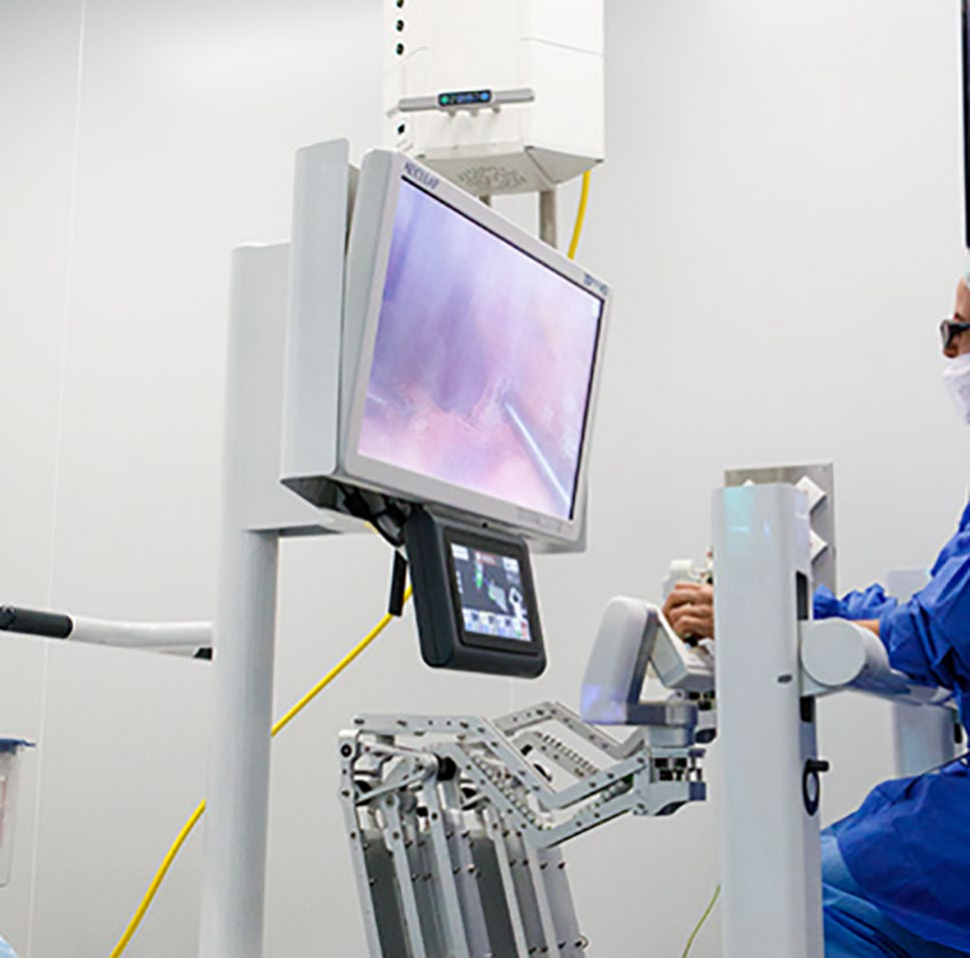
Rob Surgical Systems—S Bitrack System Surgeon Console

The Rob Surgical Systems robotic platform, Bitrack System, originated in Spain for use in urological, gynecological, and general surgery procedures. It is expected to obtain registration from European bodies in 2024 [36]. The platform consists of an open console and a multi-arm operating unit. The operating unit features four arms with passive joints and a floating fulcrum which allows free movement of the instruments without docking to trocars (Fig. 18). The architecture of the robotic column allows for multi-quadrant access while leaving an ergonomic workspace. The console features a generic 3D screen which is compatible with conventional laparoscopic cameras (Fig. 19). The Bitrack system has two passive joints which avoid excessive forces in the fulcrum point and permit natural fluctuations with patient respiration. Docking time and arm draping is reported to take less than 30 s per arm [37]. As the platform is compatible with generic trocars and available bedside operating space, surgeons are easily able to switch between robotic and manual laparoscopic procedures. RobSurgical created single-use 8 mm laparoscopic instruments featuring 7 degrees of freedom to the instrument tip with active energy options (monopolar and bipolar). Bitrack additionally features an intelligent laparoscopic navigation system and a respiratory motion compensation system available. These systems contribute to improved safety and performance by utilizing diagnostic imaging combined with real-time data for artificial intelligence [36].

### Ronovo surgical—Carina

**Fig. 20:**
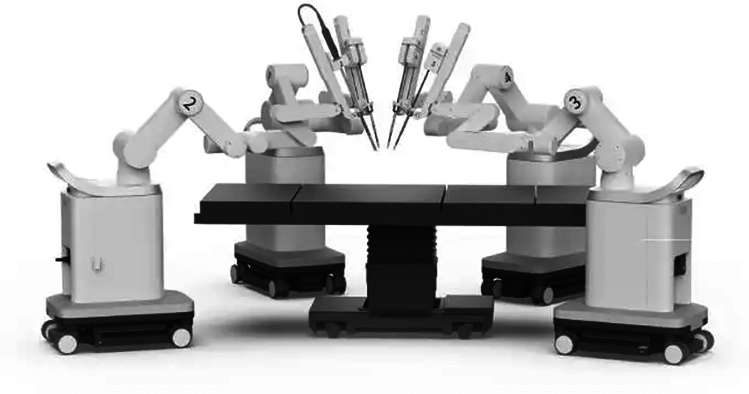
Ronovo Surgical—Carina System

The Carina Surgical platform, developed in Shanghai by Ronovo, made its debut in December 2022. It features a compact modular design with four separate carts (Fig. 20), each equipped with a robotic arm, and a closed surgeon console providing a stereoscopic immersive view. The Carina system utilizes Ronovo’s proprietary 3D HD laparoscope and a visionary system integrating fluorescence imaging and patient-specific 3D reconstruction of preoperative imaging [38]. The imaging system is also compatible with Storz (including Rubina ICG) and Olympus systems. Instrumentation includes 8 mm wristed single-use instruments, 5 mm non-wristed reusable instruments, reusable cannulas, single-use obturators, and seals, as well as typical monopolar and bipolar options. Designed for urology, gynecology, general surgery, and thoracic surgery, the Carina system achieved a significant milestone with its first human trial successfully completing a radical prostatectomy in 2023. Ronovo recently concluded a four-center, four-specialty clinical trial of the Carina platform, demonstrating zero conversions to laparoscopy or open surgery and all patients showing good recovery at the one-month mark [39]. Ronovo aims to launch Carina commercially in China in 2024, with sales model and pricing details currently unavailable [40].

### Sagebot—KangDou

**Fig. 21:**
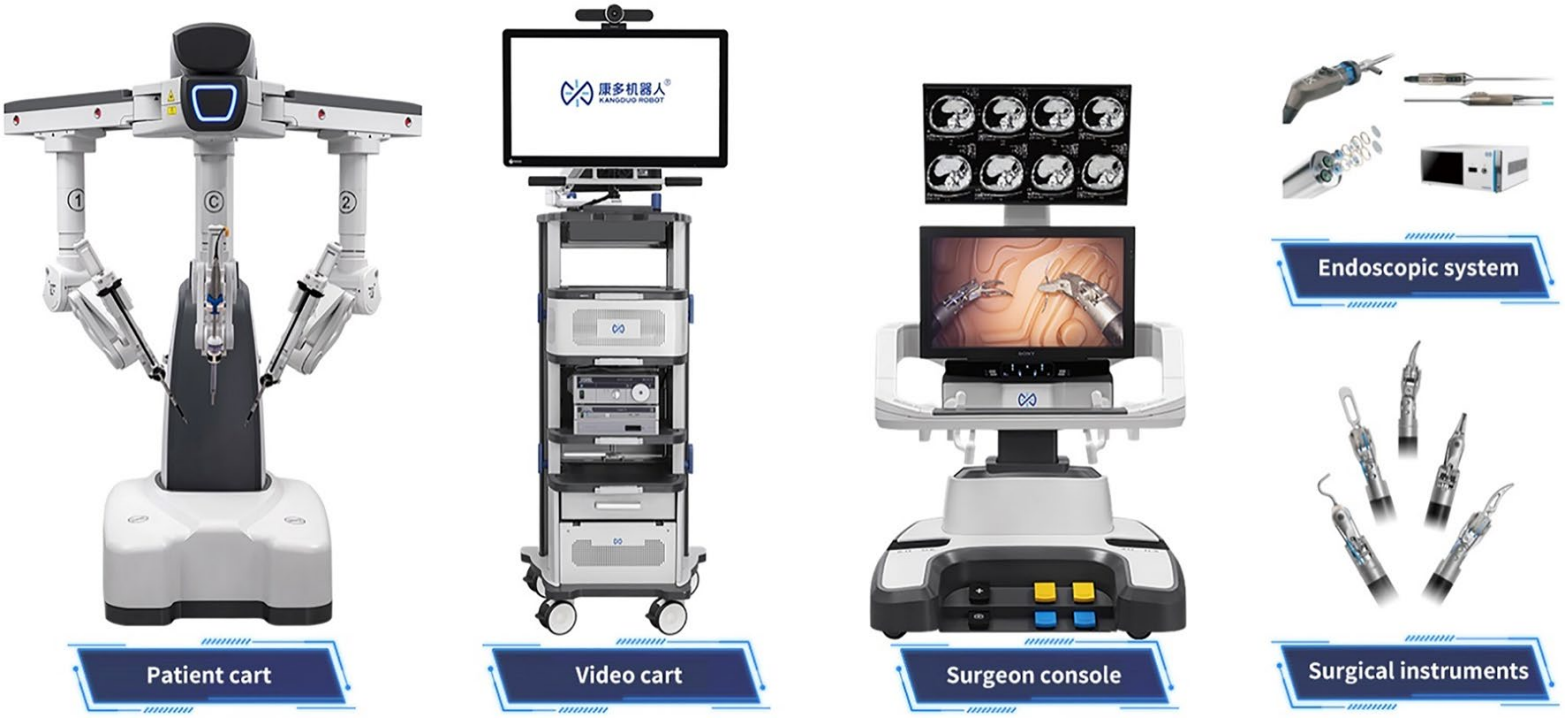
Sagebot—KangDuo

Introduced in 2020, this is a modular multi arm system with an open surgeon console (Fig. 21). The modular system currently has 3 arms but will allow for up to 5 arms with 1 scope and 4 instruments that can be engaged simultaneously. It will have dual drive capability where two operators can control two arms each at the same time to allow for actual assistance. The open console has pistol grip hand controllers and foot pedals with glasses to allow for 3d vision. 8 mm instruments (reusable up to 10 times) have 540 degrees of rotation and ultrasonic energy is available [41]. The robot was built with telesurgery capabilities. Results of early studies for prostatectomy demonstrated safety [42] and for partial nephrectomies have shown non-inferior outcomes compared to current systems [43].

### Surgerii robotic—Shurui

**Fig. 22:**
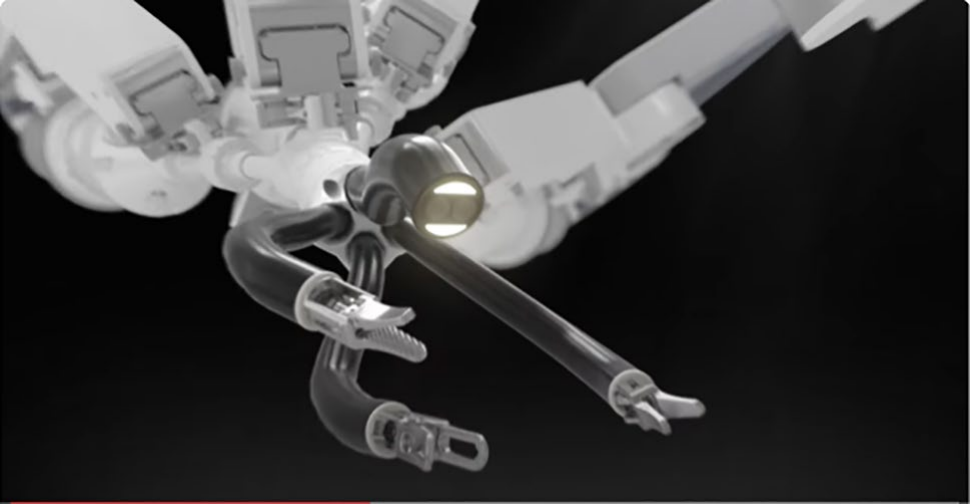
Shurui Robot Snake-like Instruments with multiple arms through a single port

This is the first single port surgical robotic system developed in China and was first exhibited in 2019. It has four arms with deformable nitinol snake-like instruments on each arm that access the body thorough a single port (Fig. 22). It has a semi-closed surgical console with hand controllers and foot pedals as well as an operating cart. It is approved in China for Urology and Gynecology and had completed trials in general and thoracic surgery [44]. Early studies have been conducted on 11 patients with prostate cancer who underwent a radical prostatectomy without need for conversion and without major intraoperative or postop complications [45]. Similar results have been noted in 63 gynecology patients across 6 academic medical center [46]. Additional single case reports of partial nephrectomy [47] total gastrectomy [48] and choledochal cyst excision [49] have been published.

### SS Innovation—mantra

**Fig. 23:**
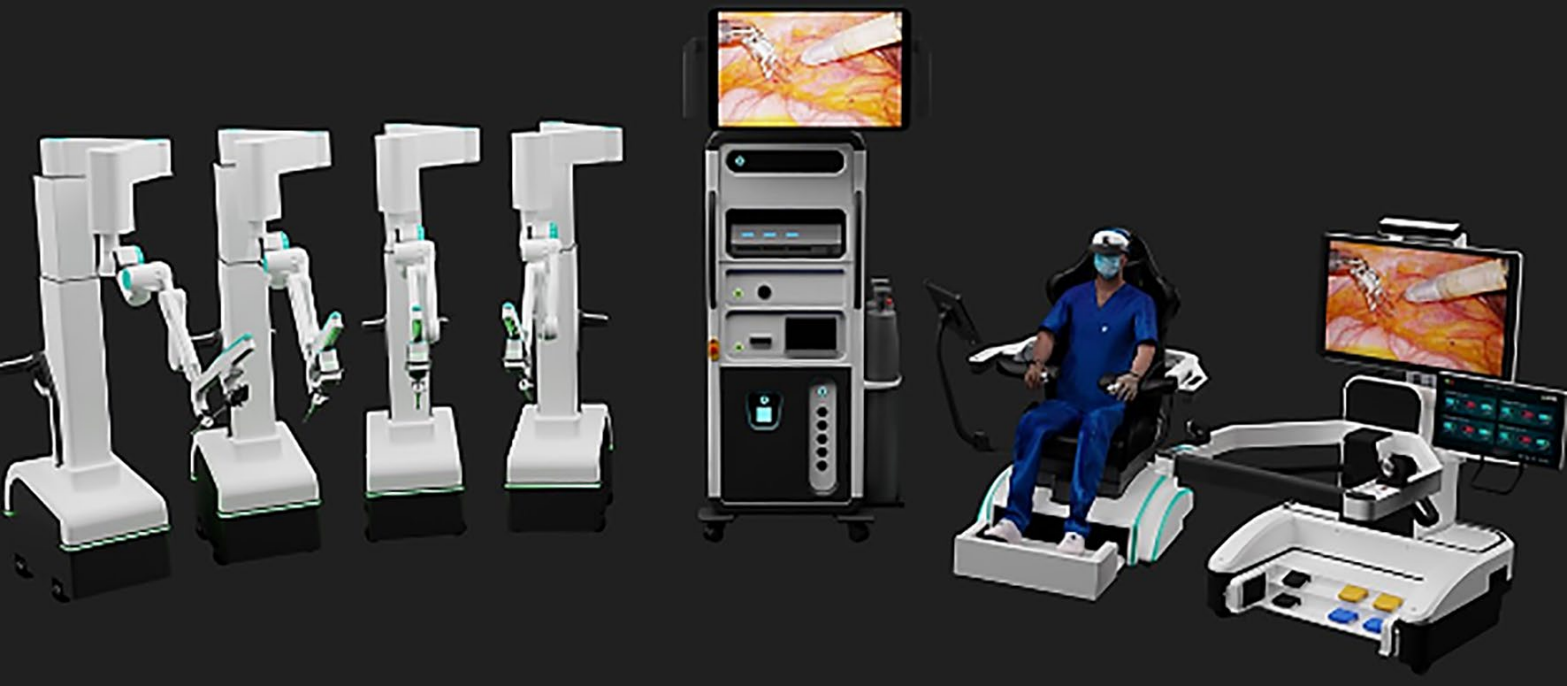
SS Innovation—Mantra Modular design

**Fig. 24:**
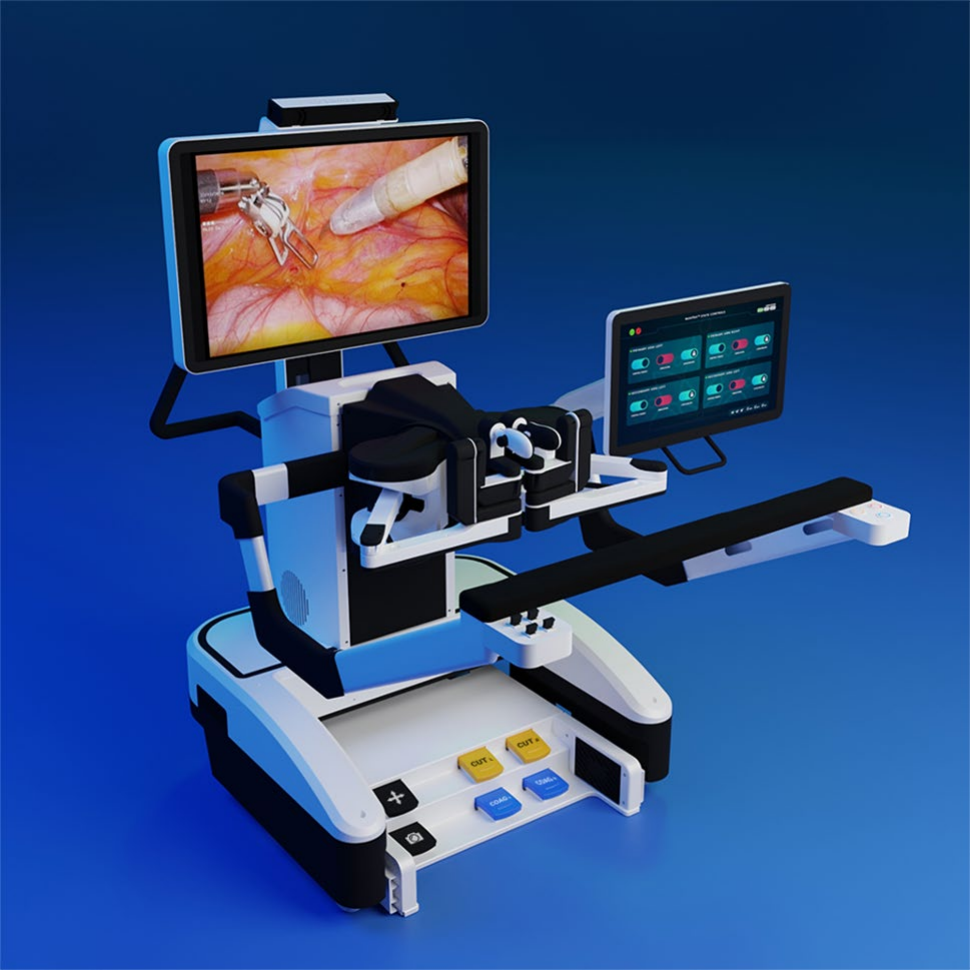
SS Innovation—Mantra surgeon console

This is a multi-arm system with five independent carts was introduced in India in 2016. The system is modular (Fig. 23) which allows for a versatile 3–5 arm configuration, enhanced portability, and its stowed dimensions measure 610 mm × 450 mm × 1640 mm. The surgeon console (Fig. 24) is open and features foot pedals equipped with arm switching, clutch, camera control, and electrocautery capabilities. Vision is 4 K-3D-HD quality, augmented by a head-tracking camera safety feature and an articulating 3D endoscope. The vision cart facilitates live streaming and recording, enabling remote training and tele mentoring. Additionally, it offers an augmented reality feature, providing intraoperative 3D holographic representations of the patient’s anatomy, seamlessly integrating accurate MRI/CT scans. The instrumentation includes a variety of more than 30 instruments most of them 8 mm (SSI MUDRA™), such as the typical monopolar and bipolar instruments, clip appliers such as medium/large Hem-o-lok ™, three different needle drivers, graspers, scissors (Potts, round tip), and several specifically designed for cardiac surgery, all of them reusable and compatible with autoclave sterilization. About to be launched is a multi-fire clip applier and a NADI-anastomotic connector for cardiac surgery. It is currently approved in India (CDSCO) for cardiothoracic surgery, urology, general surgery, head & neck, and gynecology. The Mantra robot is available in India, Sri Lanka, Nepal, Bangladesh, Indonesia, United Arabic Emirates, Ecuador and Guatemala. The CE Mark in Europe and FDA approval as well as in several Latin American countries is expected in 2024–2025.

### Titan medical—Enos

**Fig. 25:**
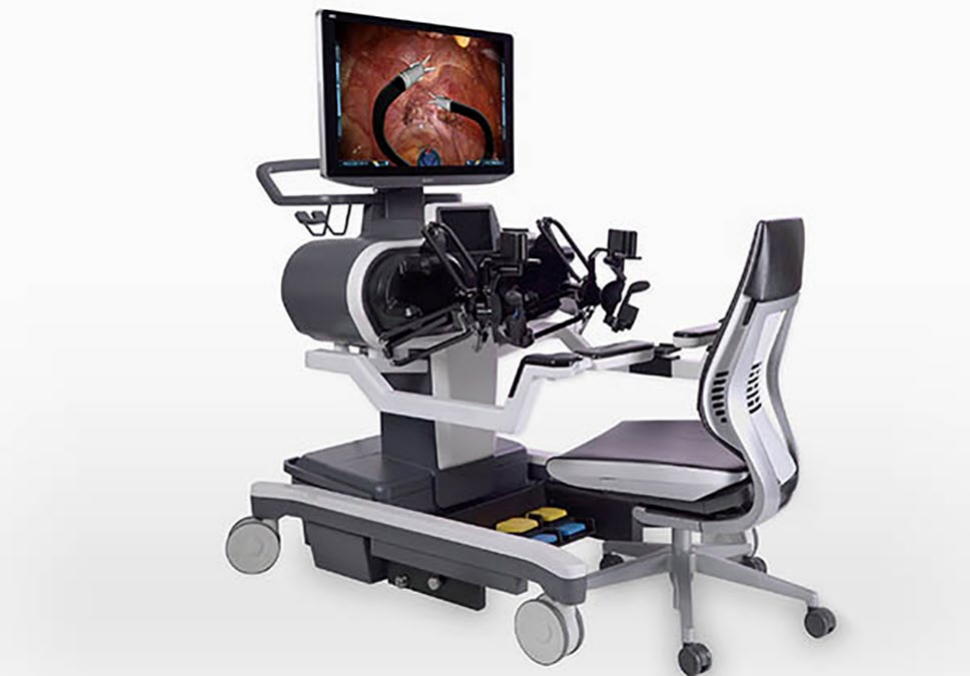
Titan Medical—Enos Workstation

**Fig. 26:**
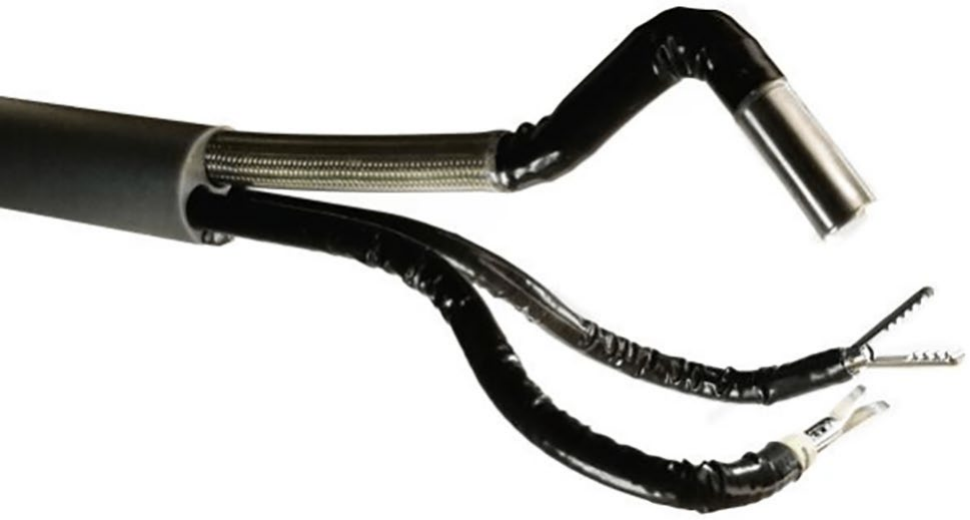
Titan Medical—Enos Arm

The Enos robot, designed by Titan Medical, is a single-access surgical system. The device, previously known as Single Port Orifice Robotic Technology (SPORT) Surgical System, was rebranded in 2020. The system is indicated for use in urology, gynecology, general surgery, and colorectal surgery. The system consists of an open surgical console (Fig. 25) and a patient cart. The Enos (Fig. 26) utilizes a 25 mm cannula inserted through a single abdominal incision, two articulating arms, a 2D high-definition, and 3D high-definition articulating camera. Enos 2.0 eliminated the 2D camera to make room for a third instrument arm. The Enos surgical robot has a suite of multiarticulate reusable wristed instruments including monopolar hooks and shears, Hunter and Maryland bipolar dissectors equipped with electrocautery, and Cold instruments such as the mega needle driver, suture cut, tenaculum, fenestrated and lap clinch effectors [50].

It is currently working towards FDA approval and has been trialed in general surgeries and colorectal procedures in cadaver models in Florida. Collaborating with Medtronic, the anticipated product launch is set for 2025 [51].

### Microport Medbot—Toumai

**Fig. 27:**
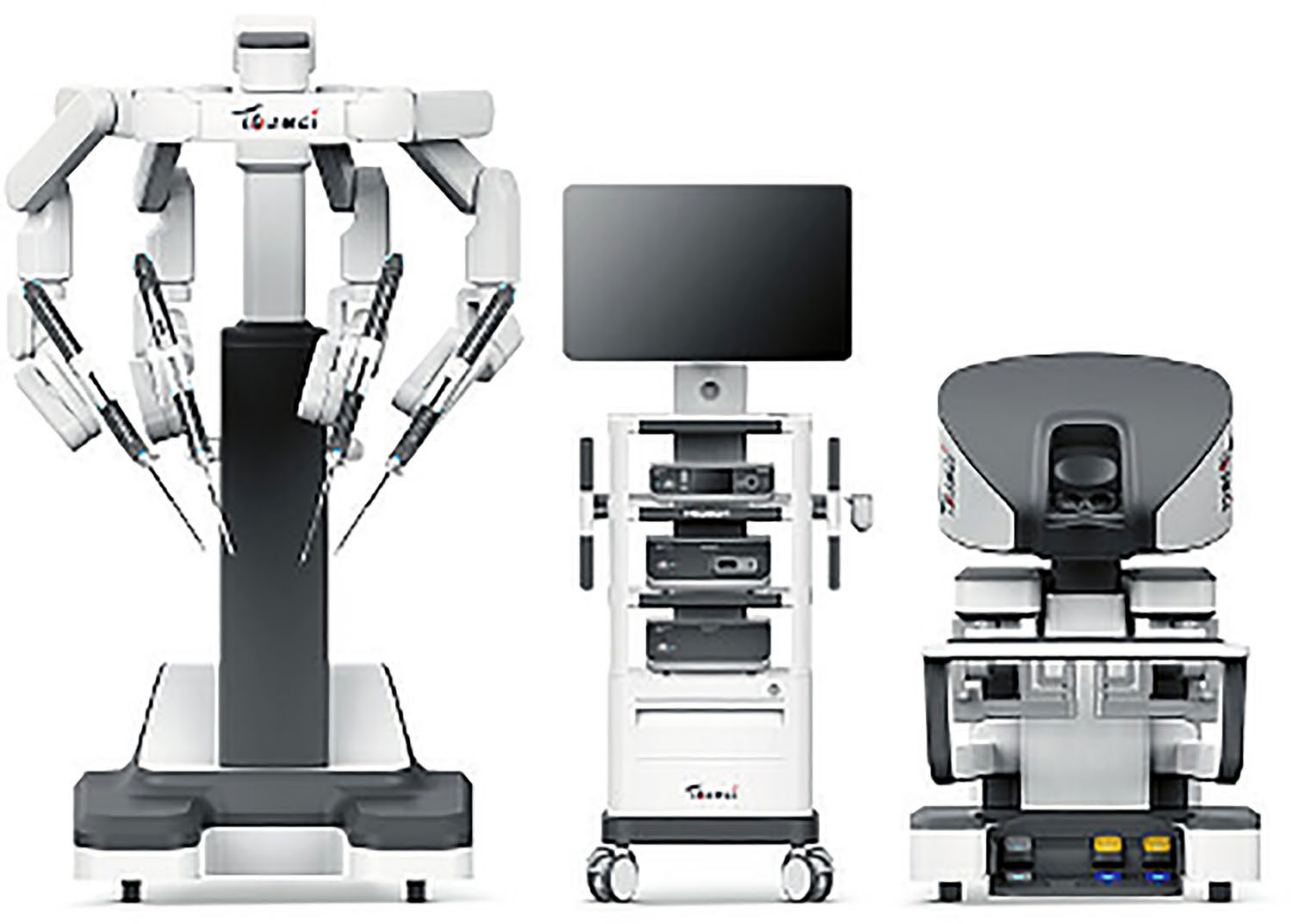
Microport Medbot—Toumai System

Microport was founded in 1998 in China and Medbot is their robot subsidiary founded in 2015. They have developed surgical robots for multiple areas including laparoscopic, transbronchial and orthopedic applications. Their laproscopic robot, Toumai, is a modular four-arm system mounted on a single patient cart with a closed surgeon console and the image vehicle (Fig. 27). It has advanced imaging with 10 × optimal magnification, instruments with 7 degrees of freedom and has force-sensing technology. It conducted its first trials in prostatectomy in 2018 [52]. Since then it has expanded use in urology, general surgery, thoracic surgery, and gynecological endoscopic surgery and has completed about 3000 surgeries [53], It was approved in China in 2022 and received EU CE certification in May 2024. It has also expanded to Africa since. The robot was built with 5 g incorporated into the design and it has demonstrated telesurgery capabilities using 5G networks been used to perform > 200 remote surgeries across > 1000 miles [54].

### Vicarious medical—Vicarious

**Fig. 28:**
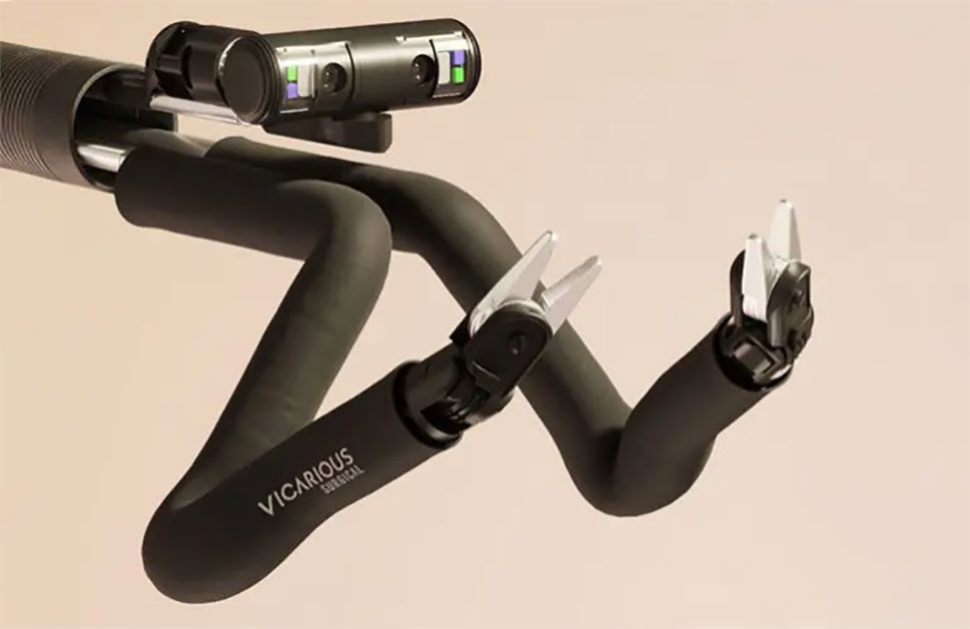
Vicarious Medical—Vicarious

Vicarious surgical is developing a platform for single incision surgery, using a 15 mm single access for intra-abdominal surgeries: GI, gallbladder, gynecology, hernias. The platform consists of an open console, and a single patient cart. The effectors and camera are all housed in a 15 mm shaft, the camera deploys along and proximal to the two effectors, each one having a shoulder, elbow, and wrist with 13 degrees of freedom per arm (Fig. 28). The company plans for FDA submission to be made in 2024–2025 through Breakthrough designation.

### Virtual incision—MIRA

**Fig. 29:**
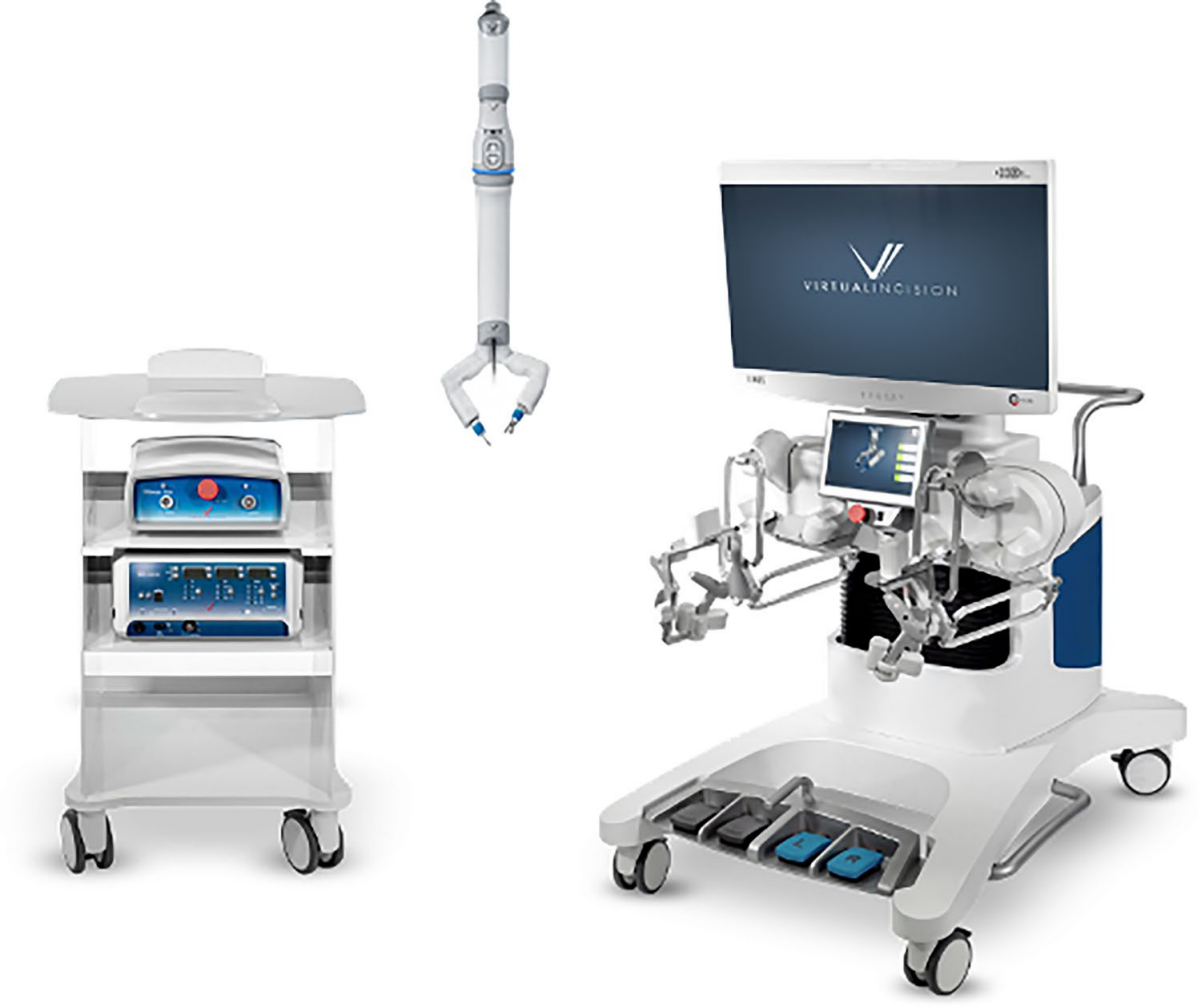
Virtual incision—MIRA

MIRA, “miniaturized in vivo robotic assistant,” was created by Virtual Incision Corporation. The device underwent the FDA’s de novo classification process and recently received marketing authorization for use in adults undergoing colectomy procedures [55]. The MIRA device features a small, self-contained surgical device inserted through a single midline umbilical incision [56]. This system (Fig. 29) consists of the MIRA robot, an open-concept surgeon console, and a companion cart. The surgeon console features two handle grips to control the instruments, two cautery foot pedals, two additional pedals for the camera and instrument clutch. The hand input devices also provide haptic feedback to indicate workspace boundaries. The current model has two 7 degrees of freedom instruments, a bipolar fenestrated grasper, and monopolar shears [3]. The device also offers internal triangulation using internal shoulders and arms, as well as infinite wrist roll [4]. The reduced size of the internal motors and pulleys allowed for the robotic arms to fit entirely inside the peritoneal cavity. Weighing a mere 2 lbs., MIRA’s design enables it to be entirely sterile processed in a standard surgical tray, eliminating the need for drapes and docks. This compact design allows for a “tray-to-table” approach, making any operating room robot ready in minutes [57].

While currently only FDA approved for colectomies, MIRA plans to broaden its indications to encompass gynecology, general surgery, urology, and various soft tissue and solid organ surgeries, with further studies in gynecology planned for 2024. Additionally, a new iteration of this technology tailored for general surgery is set to be used and studied outside the U.S. later this year [4].

### Wego—Micro Hand S

**Fig. 30:**
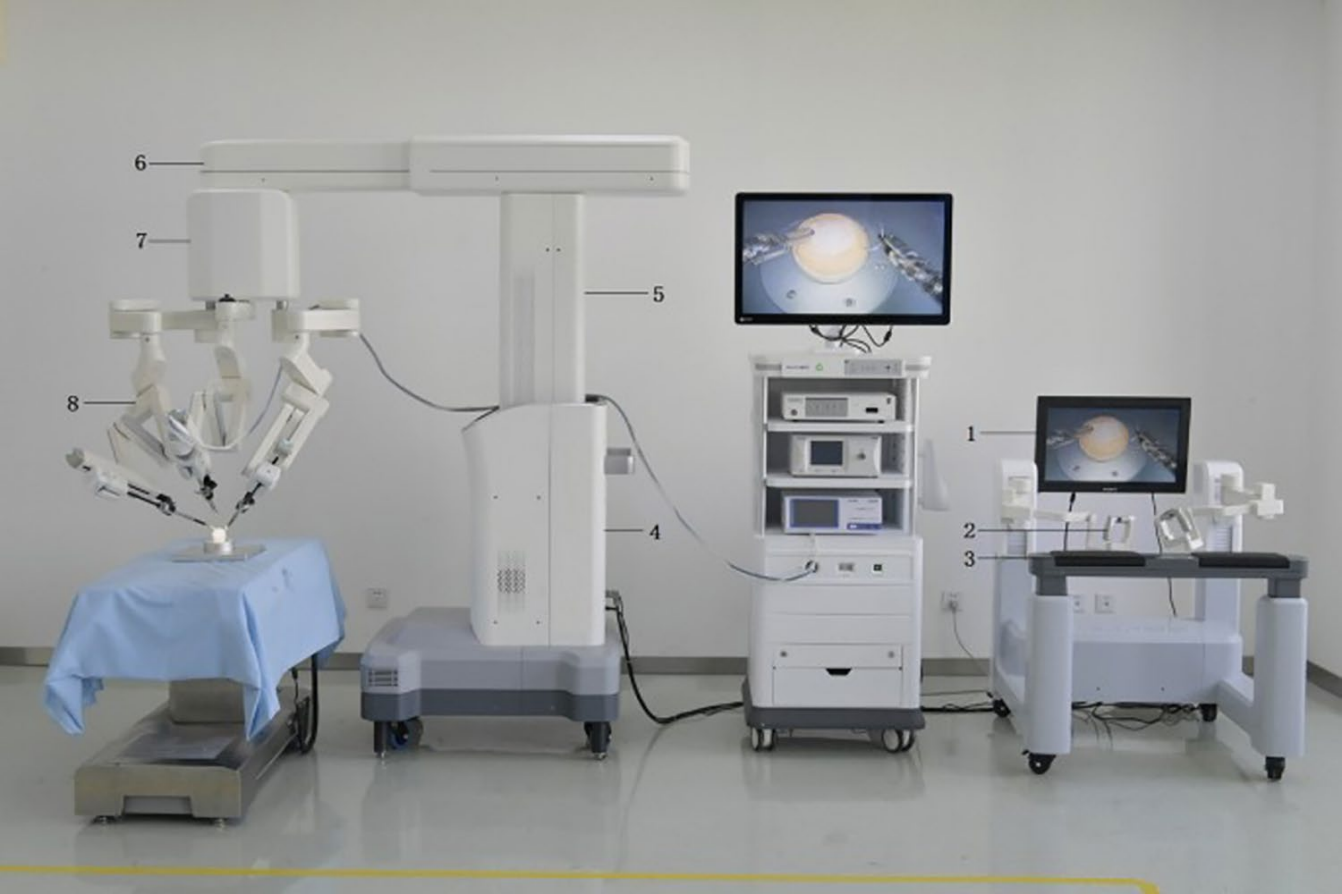
Wego—Micro Hand S System

**Fig. 31:**
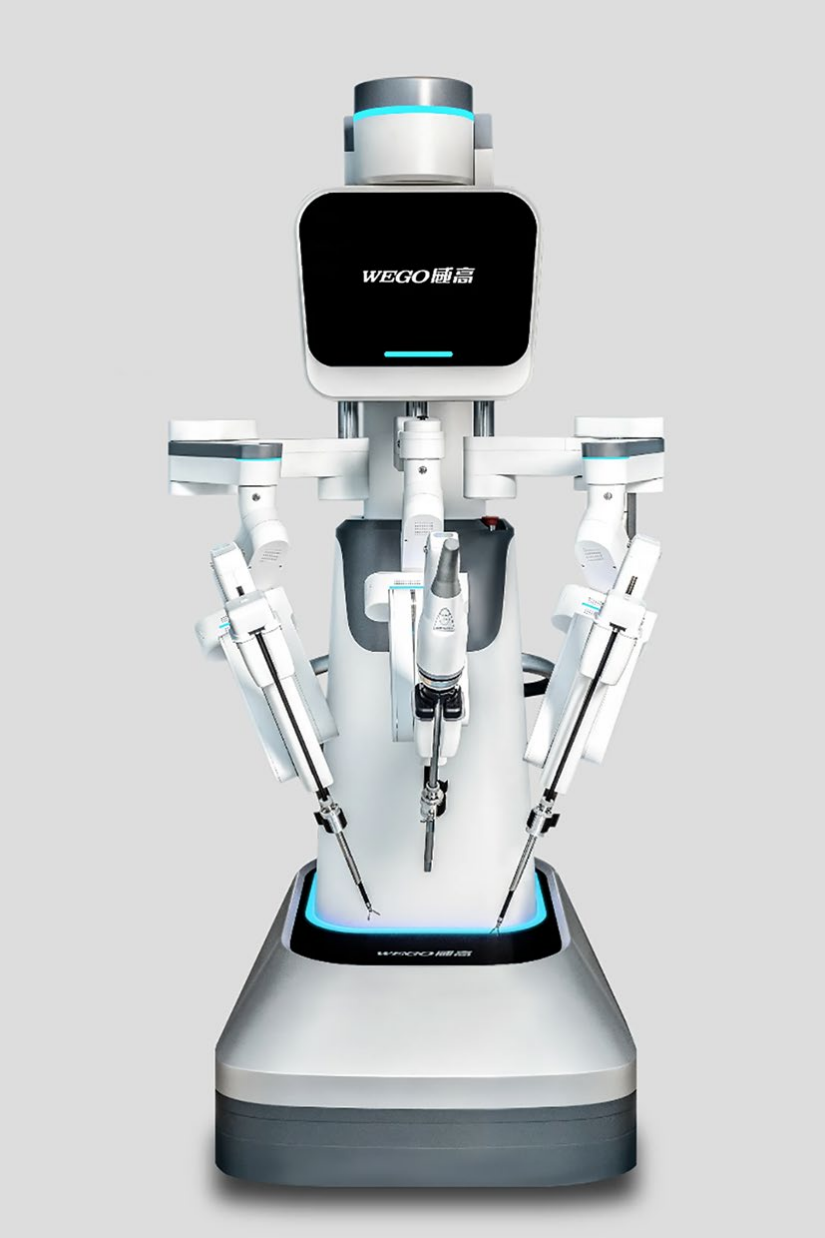
Wego—Micro Hand S Robot

The Wego-Micro Hand S surgical robot, originating from China through collaboration between multiple universities and the government, presents a multi-arm platform tailored for general surgery. Consisting of separate patient and surgeon consoles (Fig. 30), the open surgeon’s console features a base, four stand columns, armrest, two master arms, an image display device, and a control system [58]. The robotic arms are mounted on a single column moveable base (Fig. 31) helping them to consume less space [59] and employ a combination of three degrees of freedom rotation, swinging, and joint rotation [60]. The left and right operative arms can use energized instruments at the same time without influencing each other [9]. The Micro Hand S surgical robot is also equipped with a dual CMOS sensor 3D stereoscopic imaging system [61]. The 3D image produced by this imaging system is widely open so that the surgeon and others can view the screen simultaneously wearing 3D glasses throughout the procedure. The Micro Hand S surgical robot also offers adjustable action mapping – with three different options, 1:3, 1:6, and 1:10 – meaning the surgeon moving 3, 6, or 10 cm in the console will cause the operative arms to move 1 cm simultaneously [9]. Notably, the platform lacks haptics but offers 3D visualization, wristed instrumentation, and motion scaling. It also has an integrated alarm system that activates if the surgeon operates too quickly or aggressively [58].

Presently, it is primarily utilized in China, particularly in hepatobiliary, colorectal, and general surgeries. Regulatory approval status outside China remains unclear [62]. Notably, regarding sustainability, the surgical instruments of the Micro Hand S surgical robot have no limitation on the number of times they can be used [9].

## Discussion

This overview provides a comprehensive review of emerging robotic platforms in multi-visceral surgery, highlighting a variety of novel systems poised to disrupt the existing market. The rapid expansion and diversification of robotic surgical platforms signify a fundamental shift toward more versatile, cost-effective, and globally accessible solutions. A key trend emerging from the analysis is the diversification of robotic platforms, each with distinct architectures and innovative features. The platforms range from single-arm to multi-arm systems with ranges in size and mobility to optimize OR space. Such diversity reflects a deliberate effort to cater to specific surgical needs and reduce the technological footprint in operating rooms. Telesurgery is likely to be of increasing significance in the future. The first telerobotic surgery was a cholecystectomy in 2001 in France [63]. Recently there has been a resurgence in interest and in 2019, telerobotic spinal surgeries based on 5G network were performed on 12 patients in six hospitals from six different cities in China. These 12 cases showed it is possible to provide minimal latency, high bandwidth, and reliable communication for medical services with no telecommunication error or network delay [64]. In October 2023, the Hinotori platform was used to complete a remote surgery demonstration with the surgeon cockpit located in Singapore and the operating unit in Nagoya, Japan, approximately 5000 km apart [28]. The role of artificial intelligence (AI) is emerging, as seen with Moon Surgical’s Maestro and its embedded AI automatic camera control. However, most systems remain surgeon-controlled, emphasizing precision, dexterity, and ergonomics while offering advanced control features like haptic feedback and eye-tracking.

Navigating regulatory approvals remains a critical challenge for new entrants with most aiming for FDA approval in 2024–2025. European CE marks provide earlier market penetration opportunities for systems that are not of North American Origin. For this review, an attempt was made to contact the individual robotic companies, but a response was not always received.

While the reviewed platforms present significant advancements, their specific clinical applications and long-term roles within the minimally invasive surgical ecosystem remain to be fully understood. The competition is expected to drive down costs, making robotic surgery more accessible globally. Systems like SS Innovation’s Mantra and Wego’s Micro Hand S are designed for emerging markets, offering significantly lower price points. Open consoles and improved ergonomic designs may encourage wider adoption among surgeons. Systems like Distal Motion’s Dexter and CMR Versius prioritize intuitive interfaces resembling laparoscopic instrument grips, easing the transition for experienced laparoscopic surgeons. In addition, enhanced data capture and integration with hospital IT (Information Technology) systems could offer improved patient outcomes through better surgical planning and postoperative analysis.

## Conclusion

The rapid evolution and diversification of robotic platforms promise an exciting era for minimally invasive surgery. The platforms discussed in this paper collectively represent the forefront of innovation, with a clear emphasis on improved dexterity, visualization, and affordability. Future research should focus on comparative clinical trials and real-world cost-effectiveness analyses to establish each platform’s optimal role in surgery.
